# Power Laws for Heavy-Tailed Distributions: Modeling Allele and Haplotype Diversity for the National Marrow Donor Program

**DOI:** 10.1371/journal.pcbi.1004204

**Published:** 2015-04-22

**Authors:** Noa Slater, Yoram Louzoun, Loren Gragert, Martin Maiers, Ansu Chatterjee, Mark Albrecht

**Affiliations:** 1 Gonda Brain Research Center, Bar-Ilan University, Ramat Gan, Israel; 2 Department of Mathematics, Bar-Ilan University, Ramat Gan, Israel; 3 National Marrow Donor Program, Minneapolis, Minnesota, United States of America; 4 School of Statistics, University of Minnesota, Minneapolis, Minnesota, United States of America; Imperial College London, UNITED KINGDOM

## Abstract

Measures of allele and haplotype diversity, which are fundamental properties in population genetics, often follow heavy tailed distributions. These measures are of particular interest in the field of hematopoietic stem cell transplant (HSCT). Donor/Recipient suitability for HSCT is determined by Human Leukocyte Antigen (HLA) similarity. Match predictions rely upon a precise description of HLA diversity, yet classical estimates are inaccurate given the heavy-tailed nature of the distribution. This directly affects HSCT matching and diversity measures in broader fields such as species richness. We, therefore, have developed a power-law based estimator to measure allele and haplotype diversity that accommodates heavy tails using the concepts of regular variation and occupancy distributions. Application of our estimator to 6.59 million donors in the Be The Match Registry revealed that haplotypes follow a heavy tail distribution across all ethnicities: for example, 44.65% of the European American haplotypes are represented by only 1 individual. Indeed, our discovery rate of all U.S. European American haplotypes is estimated at 23.45% based upon sampling 3.97% of the population, leaving a large number of unobserved haplotypes. Population coverage, however, is much higher at 99.4% given that 90% of European Americans carry one of the 4.5% most frequent haplotypes. Alleles were found to be less diverse suggesting the current registry represents most alleles in the population. Thus, for HSCT registries, haplotype discovery will remain high with continued recruitment to a very deep level of sampling, but population coverage will not. Finally, we compared the convergence of our power-law versus classical diversity estimators such as Capture recapture, Chao, ACE and Jackknife methods. When fit to the haplotype data, our estimator displayed favorable properties in terms of convergence (with respect to sampling depth) and accuracy (with respect to diversity estimates). This suggests that power-law based estimators offer a valid alternative to classical diversity estimators and may have broad applicability in the field of population genetics.

## Author Summary

The distribution of haplotypes and species tend to be heavy tailed. The heavy tail is expected from theoretical considerations and is observed in most populations. Accurate measures of diversity are difficult to achieve given that a limited number of common haplotypes represent the majority of the population, whereas the major contributor to haplotype diversity comes from unique haplotypes that are “rare” and present in only a fraction of the population. A major issue for unrelated HSCT donor registries is estimating population coverage with respect to servicing the public need. We here use a power-law methodology that accommodates heavy-tails to estimate both the population coverage by ethnicity in the US and the genetic diversity of alleles and haplotypes. For the European American population, which has the deepest sampling amongst ethnicities, we show that registry population coverage is better than 99%, but the diversity of this sample only represents 40% of the unique haplotypes expected to be found in the population. Population coverage for other ethnicities was poorer and ranged down to 92% as was the case for Native Americans that had the worst coverage. We further show that the formalism developed here produces better estimates of the population properties than existing methods.

## Introduction

Allele and Haplotype diversity are fundamental properties in the domain of population genetics for describing the general characteristics of any population of diploid organisms. In the context of hematopoietic stem cell transplant (HSCT) matching, proper estimates of allele and haplotype diversity are essential for describing the population genetics that govern the clinical suitability of a donor/patient match. Matching algorithms scan large unrelated donor registries to identify suitable matches based upon individual Human Leukocyte Antigen (HLA) genetic typings. HLA is defined by the super locus of genes contained in the major histocompatibility complex (MHC) on the 6^th^ chromosome that encodes proteins governing the adaptive immune system response [1]. Successful transplantation requires careful matching of donor/recipient HLA to avoid adverse immune reactions that result in graft rejection or graft versus host disease [2,3]. The ambiguous nature of HLA typing, however, presents challenges for transplant matching, given that donor registries contain a range of typing methods and allele definitions that have evolved since the 1980’s. This “mixed resolution” data contains uncertainties regarding the exact alleles each donor has and their phase on the chromosome. The standard approach for resolving these uncertainties is to impute each donor’s HLA typing to a common allele resolution and set chromosomal phase using expectation maximization (EM) algorithms [4]. Given a proper candidate set of haplotypes, EM algorithms work well to estimate the distribution of this defined population, which becomes the reference data for computing accurate donor/patient match predictions.

However, we often do not know what constitutes a complete or reasonable candidate set of haplotypes describing the clinically relevant variation among individuals. This problem arises due to the following main reasons: **Lack of Adequate Sampling Depth—**the MHC region has the highest allele diversity within the human genome, so even a large sample relative to the population is unlikely to observe all clinically relevant haplotypes. For example, in the European American population 44.65% of haplotypes in the Be The Match registry are singletons (i.e. are represented by only one individual), yet the most frequent haplotype represents 6.11% of the sample so one might falsely conclude that variation is lower than it really is. This motivates the need to develop reliable methods for assessing sampling depth and the benefits of continued sampling; **Ambiguous Nature of HLA Typing—**true haplotype variation is confounded by ambiguous typing methods, which describe each individual as a set of possible alleles at each loci (consistent with HLA typing). When candidate haplotypes are constructed from cross-combinations of these possible alleles, an intractably large set results due to the “curse of dimensionality”[5]. Currently, this exhaustive set of haplotypes is heavily trimmed prior to estimation with the EM algorithm, but the trimming strategies are not quantitatively informed; **Variation in Diversity Among Populations—**ancestral migration patterns have resulted in different patterns of allele and haplotype diversity among ethnic groups, implying that sampling depth requirements are likely to vary by population. Clearly, there is a need to develop quantitative estimates of allele and haplotype diversity.

More formally, let **A** denote the number of unique HLA alleles in a population and **H** the number of unique HLA haplotypes (see Table 1 for all symbols used). Estimating **A** and **H** is a difficult problem given that we do not directly observe all alleles and haplotypes in the population. Instead, we must establish and extrapolate a relationship between the number of kinds we observe and the frequency of each kind. Mathematical frameworks for describing this relationship have been developed in the study of species richness and occupancy. Capture-recapture methods have been adapted from ecology to genetics for estimating the total number of different alleles/haplotypes [6]. Such methods could be applied to estimate **A** and **H** by tagging resamples of alleles and haplotypes. Although interesting, these capture recapture methods require a very large sampling depth for heavy tailed distributions, as will be shown. Occupancy distributions [7]offer a more natural representation of our data sampling process and use the mathematical concept of regular variation to extrapolate **A** and **H** using a power law function that describes the distribution of allele and haplotype frequencies in the population [7,8]. The power law relationship was constructed under the assumption that an infinite number of categories (or kinds) exist in the population; so for our purposes we apply a boundary constraint to estimate a finite number of categories under this general framework.

**Table 1:** List of symbols used.

Symbol	Definition
*p*_*j*_	Apriori probability of choosing a haplotype *j* for a person in the population.
*N*_totd_	Total population size.
*H* = *N*_*hap*_ = *K*_*N total*_	Number of unique haplotypes. The range of j in *p*_*j*_ is from 1 to **H**.
**A**	Total number of unique alleles in the population.
*U*(*R*)	Expected number of unique haplotypes in a sample size of R.
*α*	Power of distribution.
C	Normalization factor.
*X*_min_	Minimal value of non zero *p*_*j*_.
*X*_max_	Maximal value of non zero *p*_*j*_.
*R*	Sample size.
*Z*(*R*)	Expected probability for an unobserved haplotype in a sample of size R.
*X*_0_	Point at which slope of distribution changes.
*V*_*H*_(*x*)	Exceedance function of the haplotype frequency distribution.
*μ*_*H*_ *(x)*	Density of *p*_*j*_ values—the number of haplotypes in a region = the number of haplotypes between x and x+dx (the integral over *μ*_*H*_ *(x)* from x to infinity is *V*_*H*_(*x*)).
$p_{H\;}(x)=\;\frac{v_{H}(x)}{H}$	Density distribution function for the probability of observing a clone of frequency *x*.

In the medical field of HSCT, estimates of **A** are largely catalog based; unique HLA genomic sequences are stored in databases as alleles using locus-specific nomenclature (the best known being the IMGT/HLA database cataloging 10,653 allele definitions at the time of writing [9]). Further information on the prevalence of specific HLA alleles in the general population is provided by the Common and Well Documented (CWD) allele list [10]. Estimates of **H** are typically based on EM derived HLA haplotype frequencies, which represent nomenclature-based allele combinations that are likely to be found in the population. These estimates take into account that genetic information is inherited in blocks making the true value of **H** much smaller than the expansion of all possible allele combinations. For example, estimates of **H** within the United States population in 2013 identified over 160,000 unique 6-locus haplotypes in the Be The Match Registry, where 99% of the time **H** was comprised of combinations of “common” alleles and roughly 90% of the known IMGT documented alleles (**A**) were absent from the haplotype frequencies [11]. Thus, current methods for estimating of **A** and **H** rely upon direct observation of a given allele or haplotype. For estimating the “common” portions of **A** and **H**, this is a reliable strategy for sufficient observational data is likely to exist. However, such an approach is unsuitable for estimating the “rare” portion of **A** and **H** lacking direct observational data. Evidence for the existence of a large number of “rare” alleles and haplotypes in the overall population is outlined by Klitz [12], who modeled the effects of gene conversion, point mutations, and recombination to establish the heavy tailed nature of allele and haplotype distributions.

To estimate **A** and **H**, we, therefore, construct a heavy tail relationship based upon occupancy distributions and the properties of regular variation. This power-law framework is useful for modeling the probability mass (and number) of **A** and **H** that are unseen in the sample and represented by the “invisible” tail of the distribution. We perform this on 6-locus HLA data (A~C~B~DRBX~DRB1~DQB1; where DRBX = DRB3/4/5) from the Be The Match Registry, which is one of the largest data sets characterizing human HLA population genetics containing the typing information for 6.59 million donors [11]. We also assess registry coverage, which is the proportion of the total population occupied by the observed haplotypes, of **A** and **H** with respect to the potential base of patients seeking HSCT to inform donor recruitment strategies. Last, we discuss broader applications of the power-law methodology outside HSCT for modeling species richness in the field of ecology, which is characterized by similar heavy-tailed distributions.

## Results

### Haplotype Distribution Formalism

In order to estimate the total number of unique haplotypes **H** having probability *p*_*j*_ for haplotype j 1≤*j*≤*H*,0≤*p*_*j*_≤1, we define a counting function (*v*_H_ (*x*)),also denoted an exceedance function, representing the number of haplotypes with a relative frequency (i.e. *p*_*j*_) above or equal to *x*. Observations suggest that *v*_H_ (*x*) can be approximated by a scale free function:

$$v_{H}(x)\propto x^{-\beta }$$ (1)

Note that in reality, *v*_*H*_ *(x)* can only take discrete values. However, we here take a continuous approximation of.*V*_*H*_ (*x*) This approximation stops being valid at some maximal and minimal haplotype relative frequencies (*X*_min_ and *X*_max_) Obviously, a (unique) haplotype cannot have a frequency of less than 1 and, thus, a relative frequency of less than1 / *N*_*total*_ (*X*_min_ ≥ 1 / *N*_*total*_), where *N*_*total*_ is the total number of haplotypes in the population (twice the total population size since each sampled person has two sets of haplotypes). *N*_*total*_ is typically much larger than the number of unique haplotypes, **H.**

Similarly, above a given relative frequency, *v*_*H*_ (*x*) becomes smaller than 1, and the approximation stops being valid. The total number of unique haplotypes in this formalism is *H* = *V*_*H*_ (*X*_min_).

Given the continuous formalism, one can also define a haplotype count density (which is not a formal density function, since it is not normalized to 1), representing the number of unique haplotypes **H** in a given relative frequency interval of *dx*:

$$\mu_{H}(x)dx=v_{H}(x)-v_{H}(x+dx)$$ (2)

Based on the definition of *v*_*H*_(*x*), *μ*_*H*_(*x*) obeys:

$$\int_{x_{\min}}^{x_{\max}}\mu_{H}(x)dx=H$$ (3)

Moreover, if *v*_*H*_ (*x*) is a scale free function, so is *μ*_*H*_ (*x*):

$$\mu_{H}(x)dx\propto x^{-\alpha }dx,\text{ where }\alpha \text{=}\beta +1$$ (4)

*μ*_*H*_ (*x*)is not a probability density function (PDF). However it can be normalized, by defining:

$$p_{H}(x)=\frac{\mu_{H}(x)}{H}$$ (5)

which is a proper density distribution function. Thus, *p*_*H*_(*x*) provides a description of how common (i.e. the density) each *p*_*j*_ is in the population. Further, a proper cumulative distribution function (CDF) for the *p*_*j*_’*s* (the relative haplotype frequencies) can be derived by normalizing *V*_*H*_(*x*) where $\text{Pr(}p_{j}\leq x)=1-v_{H}(x)/H$, since $v_{H}(x)/H$ represents the complementary CDF (i.e.1-Pr(*p*_*j*_≤*x*)). Also, as noted above, the power law exponent of *μ*_*H*_(*x*) is 1 unit greater than the power law exponent of *V*_*H*_(*x*)since these two quantities share the properties linking CDFs to PDFs, namely that a PDF is proportional to the first derivative of its CDF.

Previous models of haplotype frequency using slowly varying distributions assumed unlimited population size and the existence of an infinite number of unique haplotypes **H** [7,8,13,14]. In order to estimate the properties of the real population haplotype distribution, a parallel theory is required for finite populations. The finite population affects the upper and lower bounds of the distribution. The lower bound is determined by the absence of relative frequencies smaller than 1 / *N*_*total*_ and is common to all types of distributions. The upper bound is specific to heavy tail distributions. In such distributions, the contribution of the right tail to the overall probability mass is considerable. As such, the normalization constant of the distribution is greatly affected by the maximal relative frequency in the population (*X*_max_).

We here expand these methods to finite sized populations (and not only finite samples of an infinite population). In order to incorporate the finite size of the population, minimal and maximal relative haplotype frequencies are defined (*X*_min_ and *X*_max_). As mentioned, the minimal frequency for a haplotype cannot be less than one person in the whole *N*_*total*_ population that carries one copy of this haplotype (*X*_*min*_≥*N*_*total*_^*-1*^). However, in many realistic cases, the scale free distribution only starts at a higher value than that.

The upper cutoff (*X*_max_) can be determined by the total population *N*_*total*_in the following way: Let us separate the values of *x* into bins. As mentioned, the minimum difference between relative frequencies is *N*_*total*_^-1^. We can thus define *N*_*total*_^-1^to be the minimal bin size. Per definition, at the largest ‘x’ value, there is typically one unique haplotype. Thus,*v*_*H*_(*x*_max_) = 1, leading to:

$$\mu_{H}(X_{\max})dx=1\Rightarrow \mu_{H}(X_{\max})\frac{1}{N_{total}}\leq 1\Rightarrow p_{H}(X_{\max})\leq \frac{N_{total}}{H}$$ (6)

Assuming *X*_max_ is an upper threshold, we have the equal sign in the equation and in this case *X*_max_ can then be numerically extracted from Eq 6. In many realistic cases, the scale free distribution has an exponential cutoff, starting at much lower values and *X*_max_ can be lower (see S2 Text).

### Estimates of H (Number of Unique Haplotypes)

The observed portion of **H** has been growing over the years, yet there is still a significant portion that remains unobserved. It is thus of interest to estimate the total size of **H**, and the relation between the sampling size and the portion of **H** that we actually observe. If the haplotype relative frequency distribution *p*_*H*_ (*x*) was light tailed, most haplotypes would have the same order of frequency. In such a case, it would have been enough to sample a limited part of the population to detect the vast majority of different haplotypes and estimate **H**. More specifically, if the sample would be at least an order larger than **H**, most unique haplotypes would be detected. However, in heavy tailed distributions (such as power-law distributions) the vast majority of haplotypes are very rare. A deep sampling is thus required for a precise estimate of **H** and a very deep sample is required to observe all unique haplotypes. On the other hand, the contribution of these rare haplotypes to the coverage is often negligible since most of the population has common haplotypes. The precise balance between these two phenomena is determined by the power exponent of the distribution (*α*).

Formally, the expected number of unique haplotypes (*U*(*R*)) in a sample size (*R*) can be estimated, assuming a truncated power law formalism as defined above, to be (see S1–S3 Texts):

$$U(R)=H-\frac{2-\alpha }{X_{\max}{}^{2-\alpha }-X_{\min}{}^{2-\alpha }}R^{\alpha -1}\left[\gamma (1-\alpha ,RX_{\max})-\gamma (1-\alpha ,RX_{\min})\right],$$ (7)

where γ is the partial gamma function. The total number of unique haplotypes can be computed to be:

$$H\;=\;\frac{2-\alpha }{1-\alpha }\;\frac{X_{max}{}^{1-\alpha }-X_{min}{}^{1-\alpha }}{X_{max}{}^{2-\alpha }-X_{min}{}^{2-\alpha }}$$ (8)

### Estimates of the Distribution Properties

The estimates above require the slope of the distribution (*α*). Many methods have been proposed to estimate this slope, including a fit of the CDF or the PDF, or a fit of the power of the Zipf plot [15]. However, such methods have been argued to be flawed, as discussed in an excellent review by Clauset et al. [16]. The same authors have proposed estimates for the slope of the scale free distribution for either continuous [17] (Eq 9) or discrete (Eq 10) distributions:

$$\alpha =1+n{\left[\sum_{j=1}^{n}l\text{n}\frac{p_{j}}{p_{\min}}\right]}^{-1}$$ (9)

$$\alpha =1+n{\left[\sum_{j=l}^{n}1\text{n}\frac{y_{j}}{y_{\min}-0.5}\right]}^{-1},$$ (10)

where *n* is the total number of observed haplotypes, *p*_*j*_'s are the haplotype relative frequencies, *p*_*min*_ is the relative frequency of the rarest observed haplotype, *y's* are the parallel values in absolute frequencies for the discrete case and *y*_min_ is the minimal absolute frequency.

**Fig 1:**
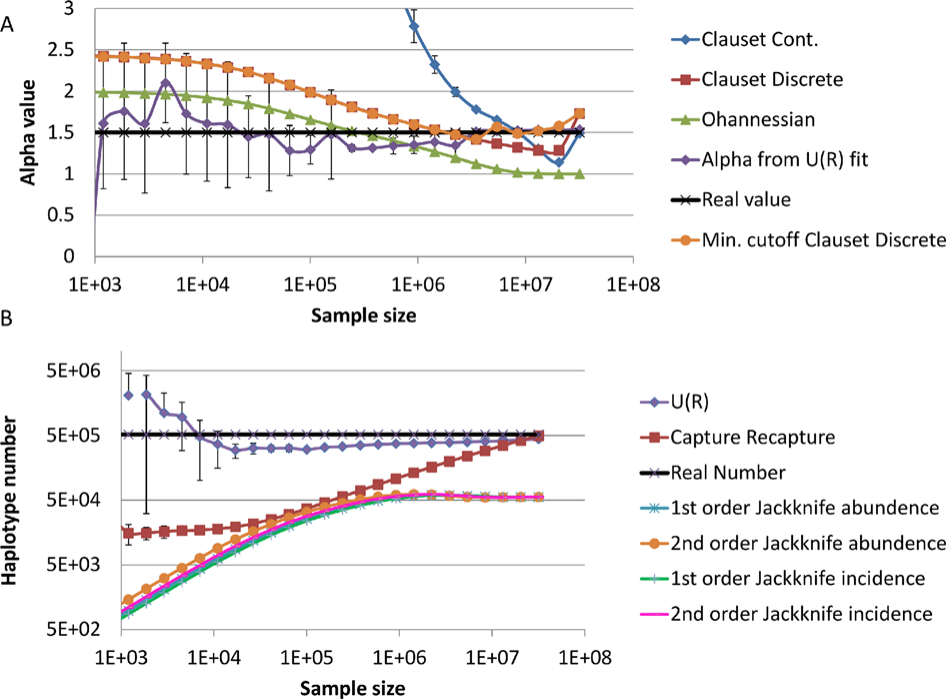
Estimation of *α* and of haplotype number for different sample sizes. **A.** Convergence of estimates of the power of distribution *α* to real value in samples from a simulated scale free distribution with a lower cutoff. The real value is the black line with the 'x' signs. The estimate using our method quickly converges to the proper value—1.5 (purple line with diamonds). The estimates using either the discrete (red line with squares) or continuous (blue lines with circles) Clauset estimates, or the Ohannessian et al. estimate (green lines with circles) do not converge, even when more than half the distribution is sampled. A Clauset discrete estimate with a minimal cutoff (orange line with circles) converges almost as well as our algorithm. **B.** Comparison of haplotype number estimate as a function of the sample size, using a capture recapture method (red squares), Jackknife estimators and the parametric estimate proposed here (blue diamonds), using the same simulation as above. The real number is a black full line with 'x' signs. One can clearly see that the parametric estimate developed here converges to a good estimate, even for a very small sample.

Another estimator based on the asymptotic properties of regular variation was proposed by Ohannessian et al. for *α*∈[1,2] [7,18]:$\alpha =\frac{K_{n,1}}{K_{n}}+1$. This estimator is based on the ratio between *K*_*n*,1_—the number of haplotypes which appear only once in the sample, and *K*_*n*_—the total number of unique haplotypes in the sample. Note that this estimator was derived for the CDF and *β* = *α*-1, which is 1 less than our PDF based estimator. While these methods are precise for a full power law distribution, they do not converge to the proper value when the distribution is truncated either from above or from below (Fig 1A). We also checked the convergence of Eq 10 when using minimal absolute frequency y*which is larger or equal to *y*_min_mentioned above:$\alpha =1+n{\left[\sum_{j=J_{\min}}^{n}l\text{n}\frac{y_{j}}{y*-0.5}\right]}^{-1}$. This truncated power law estimate does converge to the right value, but very slowly (Fig 1A orange circles). Formally, we estimated the fit to the distribution using a Kolmogorov Smirnov test, and used the lower cutoff *J*_min_ producing the best fit.

**Fig 2:**
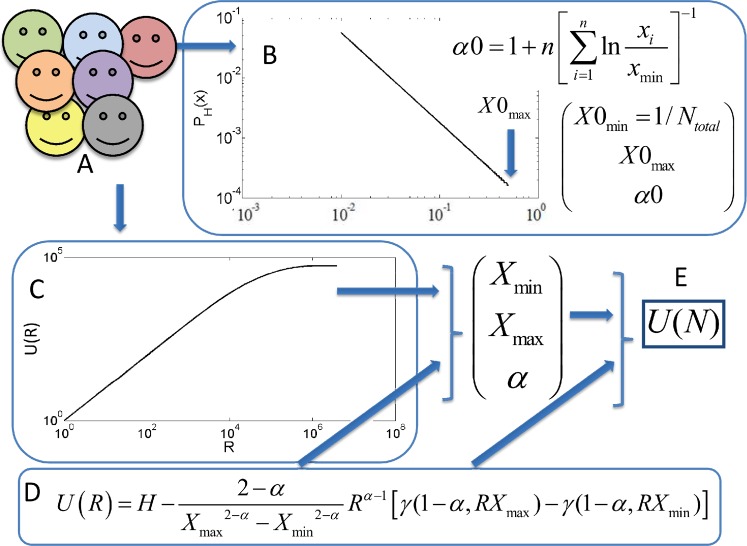
Schematic figure. (A) We observe a population with different haplotypes. From the population we extract two measures—the haplotype frequency distribution (B) and the number of unique haplotypes as a function of the sample size (C). We assume that the frequency distribution (B) is a scale free distribution with upper and lower cutoff values *X*_min_≤*x*≤*X*_max_. For the sake of simplicity, we assume a zero probability to observe haplotypes with frequencies beyond these values. We provide an initial guess for the lower cutoff to be limited by the total population size, and the upper cutoff, we limit by an upper estimate from the total population size (Eq B6). We use an initial guess of *X*_min_ at its boundary, the Clauset estimate for the slope, and the observed highest frequency *X* 0 _max_. We then fit the observed unique haplotype curve (C) with an analytical formula for the expected shape (D) with a cost function of $\sum_{\text{R'}}{\left(\log(\text{U(R')-log(observed (R')}\right)}^{2}*\log(\text{observed (R'))}$ for different values of sample sizes R'. Finally, we extract the optimal parameters (E) and produce an estimate of the number of unique haplotypes for any population size N, where N is the target population size.

We have thus adopted a different approach based on a numerical fit of the observed value of *U*(*R*) to Eq 7–8 for different values of R. We compute *U*(*R*)from the observed data for different *R* values and find the parameters leading to a best fit between Eq 7–8 and the observed data. The parameters are bounded by Eq 6 for *X*_max_ and *N*_*total*_^-1^ for *X*_min_. The numerical minimization is initialized with *X*_max_ from the observed data, *X*_min_ at its boundary value, and *α* as it is computed from Eq 9 (for a schematic figure see Fig 2). The value of *α* computed using this estimate converges to the proper value at limited sampling depth (Fig 1A).

### Methodology Validation

To validate the correctness of the haplotype number estimate in Eq 7 and 8, we did multiple validations as follows: As a first validation, we chose values of *X*_max_, *X*_min_ and *α*, and then calculated **H** using Eq 8. We generated *H* haplotypes with relative frequencies (*p*_*j*_ ’ *s*) taken from a pure power law distribution with exponent of *α*. Next, we generated samples of different sizes (R) based on the *p*_*j*_ ’ *s* values. We compared the expected value of *U* (*R*)(Eq 7) to the actual number of different unique haplotypes that we got in the sample, with an excellent fit (less than 1% deviation—S1 Fig). A similar analysis with a truncated power law gave similar results.

We then compared the convergence rate of our method with other frameworks for estimating **H**. We used a simulated population from a truncated power law, as described above and formed sub samples of incremental size. We then generated estimates of **H** as a function of increasing sub-sample size.

We compared our method with a capture-recapture formula [6]. This method was adapted to compute the total number of different alleles instead of the total population as is performed in ecology. In order to compute the capture recapture estimate, we divided each sub-sample into two smaller sub samples: *A* and *B*. We then computed the ratio between the product of the number of different alleles/haplotypes in A and B and the number of different alleles/haplotypes in their intersection:|Unique(A)|*|unique(B)|/|Unique(A∪B)|. We further compared our method with a Jackknife based estimator [19]. Capture-recapture and power-law estimates were found to converge to the true value of **H,** but the capture-recapture method required a very deep sample of the population to attain accuracy whereas the power-law method converged quickly and offered accurate estimates, even with limited sampling (Fig 1B). The Jackknife based estimator did not converge to the proper value.

**Fig 3:**
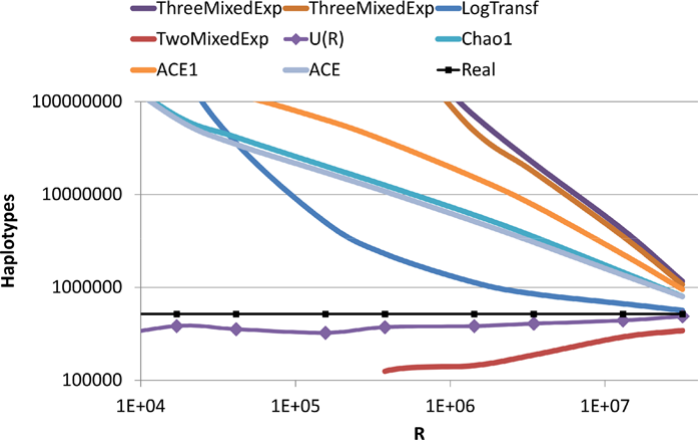
Comparisons of methods to evaluate the haplotype number. We compare the Bunge and Barger (2008) method as implemented in the Catchall model using a population with scale free frequency distribution of haplotypes with a slope of -1.5 and compare the estimated species richness as supplied by the Catchall software for all models where the software converges to a result. These models include parametric predictions procedures, such as Mixture-of-two-exponentials-mixed Poisson and Mixture-of-three-exponentials-mixed Poisson on the observed species richness or on their log values and non-parametric procedures, such as the ACE (Abundance-based Coverage Estimator) ACE1 (Abundance-based Coverage Estimator for highly heterogeneous cases) and different versions of the Chao-Bunge gamma-Poisson estimator. Each line represents the estimated number of haplotypes using one of the Catchall methods. The black line represents the real simulated species number. The purple line with diamonds represents our estimates. Our estimates converge much faster and to a much more precise estimate of the real haplotype number than any of the methods proposed by Catchall.

The field of species richness estimation is very wide and a large number of methods to estimate species richness have been proposed (e.g. [20] and supplementary material therein). Many of these methods have been merged into the CatchAll software [21]. To further compare our method, with existing state of the art methods, we have tested these measures on the same samples. All methods proposed by CatchAll showed a very slow convergence, or actually no convergence (Fig 3).

**Fig 4:**
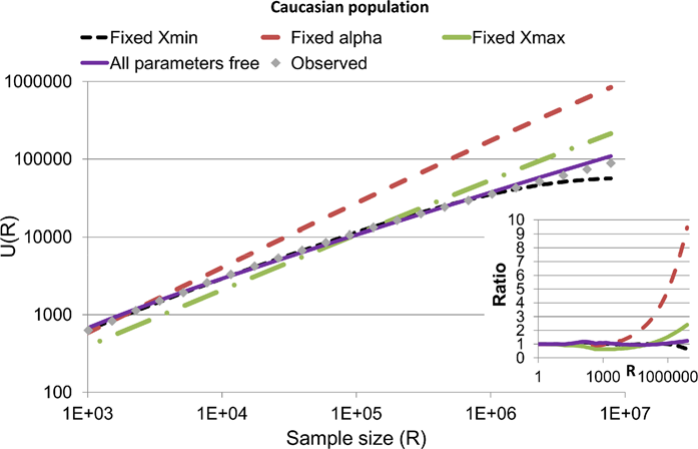
Comparison of computed and observed expected number of haplotypes in a sample of size R : *U*(*R*)for the European American population for different sample sizes R. The gray squares are observations. All estimates were performed using 10% of the sample. The lines are an estimation of *U*(*R*) with all free parameters (full purple), maximal *Xmax* and free *α* (dashed green line), free *Xmax* and free *α* but fixed *Xmin* (dashed black line) and free boundaries but *α* based on the Clauset estimate (double dashed red line). The inset shows the ratio between the computed number of haplotypes and the real one.

In order to further validate the methodology in a realistic regime, we used 7.8 million samples from Be the Match Registry, which identified 88,621 haplotypes as estimated in the EM algorithm from the European American population, and used a sub-sample of half a million haplotypes to estimate the parameters of the distribution (*X*_max_, *X*_min_ and *α*). We then validated that the resulting values fit the observed distribution all the way to the full sample size (7.8e6) and extrapolated it to the total European American population as defined by the Census [22]. The final value is an estimate of the total number of haplotypes in the European American population in the US (Fig 4; see S2 Fig for the other populations). When we estimated *X*_min_, *X*_max_ and *α* from the distribution (purple full line) an excellent fit was obtained.

To investigate the sensitivity of the model to parameter estimates of *X*_min_, *X*_max_ and *α*, we fixed the value of *α* based on the Eq 9, and optimized *X*_min_ and x_max_ or fixed one of these variables at their extreme values and optimized *α*. The fit diverges from the observed result (Fig 4 and S2 Fig). The strongest deviation is for a fixed *α*, and the smallest is for a fixed *X*_min_. A fixed *X*_min_ is expected to have the smallest effect, since its estimate is closest to the reality in the studied distributions.

To summarize, the method above has been shown to converge properly in simulated and sampled data. However, for accurate estimates, it is recommended to treat all parameters *X*_min_, *X*_max_ and *α* as free parameters to be estimated in the model.

### Haplotype Numbers in US Sub-populations

The resulting total number of haplotypes (**H**) in the European American population in the US is 286,787. Values for other sub-populations are given in Table 2. For most populations, the haplotype frequency distribution follows a power law with exponent α of the PDF between 1.4 and 1.9. Further, estimates of the power law exponent converged before the full sample size was analyzed (Table 3 and S3–S8 Figs).

**Table 2:** Analysis of the five combined populations (African-Americans, Asian and Pacific Islanders, European Americans, Hispanic and Native Americans).

Population	African Americans	Asian / Pacific Islander	European American	Hispanic	Native Americans
Size of population in Census	3.77E+07	1.49E+07	1.97E+08	4.52E+07	2.25E+06
Exponent value (Value of alpha)	1.47	1.54	1.52	1.52	1.68
Current sample size (R)	1.01E+06	1.14E+06	7.82E+06	1.43E+06	9.23E+04
Current fraction of population covered	0.983	0.985	0.994	0.984	0.934
Current number of known haplotypes	3.72E+04	3.69E+04	8.86E+04	4.45E+04	1.00E+04
**H**	1.42E+05	9.98E+04	3.16E+05	1.77E+05	4.70E+04
U(R) for current sample size	3.89E+04	3.83E+04	9.45E+04	4.73E+04	1.08E+04
U(R) for the whole population	1.30E+05	9.02E+04	2.87E+05	1.61E+05	4.17E+04
Target sample size in order to get to 99.4% coverage	4.89E+06	3.88E+06	7.76E+06	6.16E+06	1.66E+06

The first row is the total size of the sub-population in the census, the second row is the estimate of *α* from *U*(*R*). The third row is the sample size. The following rows are fraction of the population covered, current known haplotypes number, estimate of maximal haplotype number, estimate of observed haplotype number according to the *U*(*R*) estimation. The following row (eighth row) is the estimated number of haplotypes for the entire census population. The last row is the required population size to get coverage similar to the European American population.

**Table 3:** The values of *α* obtained from the 21 detailed US populations studied here.

	Number of Samples	Clauset	Estimate with half population	Estimate with full population
1. African American	8.33E+05	1.75	1.46	1.45
2. Caribbean Indian	2.87E+04	2.49	1.62	1.79
3. African	5.71E+04	2.17	1.58	1.56
4. European American	7.82E+06	1.78	1.62	1.60
5. Chinese	1.99E+05	1.96	1.59	1.58
6. South Asian	3.71E+05	1.86	1.59	1.58
7. Filipino	1.01E+05	2.08	1.60	1.59
8. South/Cntrl Amer. Hisp.	2.93E+05	1.94	1.55	1.54
9. American Indian South or Central American	1.19E+04	2.86	1.61	1.60
10. Hawaiian or other Pacific Islander	2.30E+04	2.49	1.66	1.65
11. Black South or Central America	9.78E+03	2.87	1.65	1.63
12. Alaska Native or Aleut	2.75E+03	3.27	1.79	1.77
13. Japanese	4.92E+04	2.04	1.55	1.55
14. Other Southeast Asian	5.59E+04	2.21	1.84	1.84
15. North American Indian	7.16E+04	2.12	1.53	1.52
16. Korean	1.55E+05	1.85	1.51	1.50
17. Vietnamese	8.71E+04	1.99	1.60	1.59
18. Black Caribbean	6.67E+04	2.16	1.60	1.58
19. MidEast/No. Coast of Africa	1.42E+05	2.08	1.58	1.57
20. Caribbean Hispanic	2.31E+05	1.94	1.54	1.54
21. Mexican or Chicano	5.22E+05	1.88	1.55	1.54

The second column is the number of samples used for these populations. The following columns are *α* estimates, based on the Clauset estimator (third column) and our parametric method, using half the sample or the full sample (last two columns).

An important aspect of Eq 7 is that it does not saturate until the sample size is close to the full population size. We therefore expect that haplotypes discovery rate should remain appreciable with continued sampling given that the current sample size is about 3.97% of the European American populations. In contrast, if the distribution were light-tailed, we would be expected to detect all of the haplotypes that exist. Since the distribution is heavy tailed, we estimate to have detected only 30% of **H** that exist in the US population (Table 2).

### Population Coverage

An important aspect of the low value of α (*α* ≤ 2 in most cases) is that there is a large difference between the fraction of observed haplotypes and the population coverage, defined as the fraction of samples (two per person) with a known haplotype. When α is close to 1, most of the haplotypes have practically no contribution to the coverage. Actually only very few haplotypes in the right hand tail of *v*_*H*_(*x*) (the larger values of *x*) contribute to the population coverage. For example, in the European American population, the most frequent 1% of haplotypes provide 73.18% of the coverage.

The fraction of the population with haplotypes which have already been identified can be computed to be (see S4 Text):

$$FractionCovered(R)=1-\frac{2-\alpha }{X_{\max}{}^{2-\alpha }-X_{\min}{}^{2-\alpha }}R^{\alpha -2}\left[\gamma (2-\alpha ,RX_{{}_{\max}})-\gamma (2-\alpha ,RX_{{}_{\min}})\right],$$ (11)

where γ is the partial gamma function. As was the case for Eq 7, we compared the values obtained from this calculation to the values obtained in the simulation mentioned above, and obtained an excellent fit (S9 Fig).

**Fig 5:**
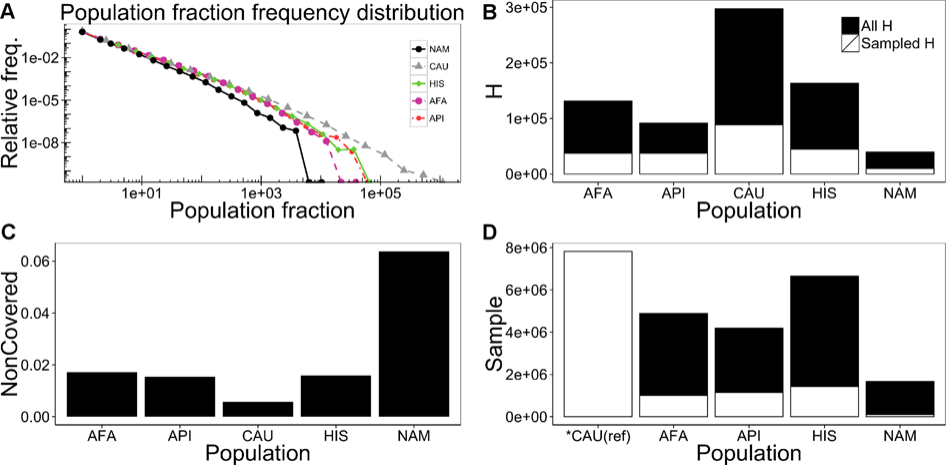
Properties for haplotypes frequency distribution for 5 populations : Native Americans (NAM), European Americans (CAU), Hispanic (HIS), African-Americans (AFA) and Asian Pacific Islanders (API). (A) Histogram of haplotype relative frequency distribution for the five combined populations: Native Americans (NAM, black circles), European Americans (CAU, gray triangles), Hispanic (HIS, green diamonds), African-Americans (AFA, purple circles) and Asian Pacific Islanders (API, red circles). (B) Estimated (black) and currently observed (white) total number of haplotypes. (C) Fraction of non-covered population for the five combined populations. (D) Current sample size (white) and estimated sample size required to get coverage similar to the European American population (black) for the five combined populations. Note that if the fit to a truncated power law would be precise, the required and observed population size would be precisely equal for the Caucasian population. However, there are some limited deviations in the fit, and the required population from the theoretical analysis is 7.76E+06, while the observed population size is 7.82E6 (a difference of less than 1%).

One can apply Eq 8 to the European American population, for example, and obtain that only around 88,620 haplotypes are identified today, out of over 286,787 haplotypes that are estimated to exist in the total population (Fig 5A and Table 2). However, the coverage of these 88,620 haplotypes is about 99.4% (Fig 5C and Table 2). The relationship between the coverage and the number of haplotypes depends on α, and varies among the studied populations. We analyzed 5 combined populations, for which clear census based estimates of the total population size are available (African-Americans—AFA, Asian Pacific Islanders—API, European Americans—CAU, Hispanic—HIS and Native Americans—NAM), and calculated the sample size needed in order to have the same percent coverage of the European American population (Fig 5D and Table 2). The combined populations have similar distributions (Fig 5A), with the European American population having the flattest distribution (least diverse, or most contribution from frequent haplotypes), and the Native American having the steepest slope (most diverse, and the least contribution from frequent haplotypes). However, since as mentioned above the number of haplotypes (**H**) is affected by the total population size, the Native American population is actually the least diverse, while the European American population is the most diverse, when **H** is taken as a measure of the diversity (Fig 5B). While most haplotypes are not sampled, most individuals in the population carry known haplotypes. As mentioned earlier, 99.4% of the European American population is covered by observed haplotypes (Fig 5C). Coverage is also more than 98% for the Hispanic, African-American and Asian/Pacific Islander populations. Coverage is less for the Native American population at 93.4% percent. In order to reach the percent coverage that currently exists for the European American population, 1 to 6 million additional donors would need to be recruited for each of the other populations (Fig 5D). This represents a factor between the required and existing sample size of around 3 for the Asian Pacific Islanders, but up to 15 for Native Americans, as summarized in Table 2.

### Probability of New Haplotype Discovery

The contrast between the small fraction of haplotypes known and the large coverage implies that the frequency of each undiscovered haplotype is expected to be very small. The probability that a haplotype from a newly sampled host is not present in the current sample of size R is (see S4 Text):

$$Z(R)=\frac{2-\alpha }{X_{\max}{}^{2-\alpha }-X_{\min}{}^{2-\alpha }}R^{\alpha -2}\left[\gamma (2-\alpha ,RX_{\max})-\gamma (2-\alpha ,RX_{\min})\right]$$ (12)

where γ is the partial gamma function. We validated the accuracy of our analytical equation using simulation (S10 Fig). This function decreases slowly as a function of sample size *R*, which explains the high degree of population coverage achieved even with limited discovery of all haplotypes in the population. Additionally, this value could be used to assign an expected frequency to missing haplotypes, which in turn could be used in HSCT matching to assign haplotype frequencies to newly discovered haplotypes not covered by the EM haplotype frequencies.

### Estimates of A (Number of Unique Alleles)

A similar analysis can be performed on the observed portion of **A**. Each haplotype is composed of alleles at 6 genetic loci: ‘A’,’B’,’C’,’DQB1’,’DRB1’,’DRBX’. We separated the haplotype data and generated allele distributions for each of the 6 loci. Our assumptions about the alleles are just the same as for the haplotypes, and a similar analysis was performed on each loci. We fit the expected number of alleles *U*(*R*) as a function of the sample size *R* to the actual number of unique alleles in partial samples of the data we have for alleles (see Methods) to obtain the values of *X*_min_, *X*_max_ and *α*.

**Fig 6:**
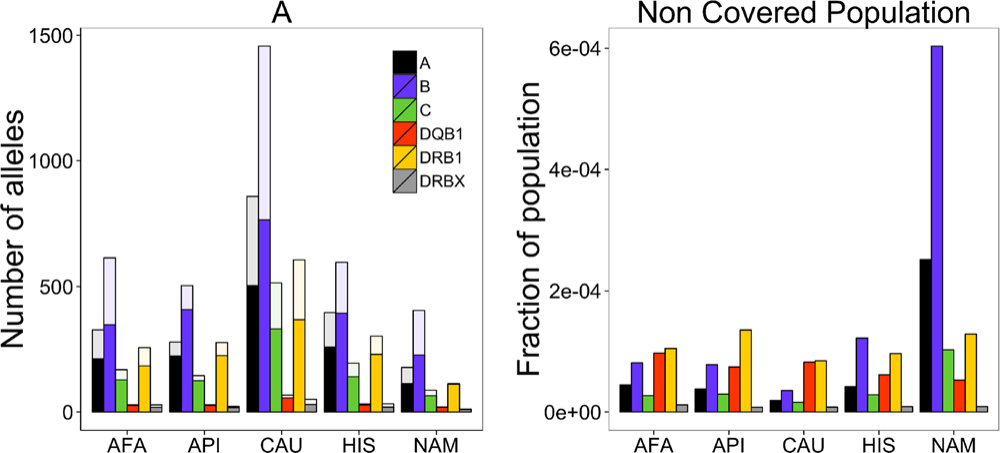
Alleles results. (left) Known (colored) vs. total alleles (transparent) for the five combined populations. The fraction of known alleles is between 50 and 100%. (right) Fraction of uncovered population per allele and combined population. These fractions are much lower than for haplotypes and never reach more than 0.1%.

The allele distribution by loci is very flat with powers approaching 1 (Table 4). Such powers give most of the importance to the frequent alleles and reduce the total number of alleles for a given population size. Indeed, estimates of **A** according to the current sample for the data of the five combined populations are not much higher than the observed number of alleles (Fig 6 left plot).

**Table 4:** Estimate of *α* for the allele frequency distribution for all allele types and for each combined sub population.

Population	A	B	C	DQB1	DRB1	DRBX
African Americans	1.19	1.21	1.17	1.01	1.05	1.03
Asian / Pacific Islander	1.15	1.17	1.16	0.93	1.09	0.99
European American	1.28	1.29	1.23	0.97	1.15	1.07
Hispanic	1.20	1.14	1.15	0.99	1.11	1.02
Native Americans	1.13	1.18	1.07	1.04	1.06	0.98

Consequentially, the fraction of uncovered population is much smaller for the alleles than for the haplotypes reaching 0.1% in the worst case (Native Americans for HLA—B which is the most diverse locus (Fig 6 right plot)). As expected by their reported diversity [9], Class I HLA alleles have a slightly higher power exponent (1.2–1.3) than class II (around 1) (Table 4). The class II power exponents, which are all close 1, are expected from neutral evolution model [23], highlighting that if selection occurs, it is mainly focused on class I or it is occurring at the haplotype level.

Surprisingly, the discovery rate for new alleles in the IMGT database appears to be increasing, in contrast with the conclusion that most alleles are known, raising the suspicion that the total number of existing alleles is much larger than current estimates. However, the estimates above propose that most alleles are currently known.

**Fig 7:**
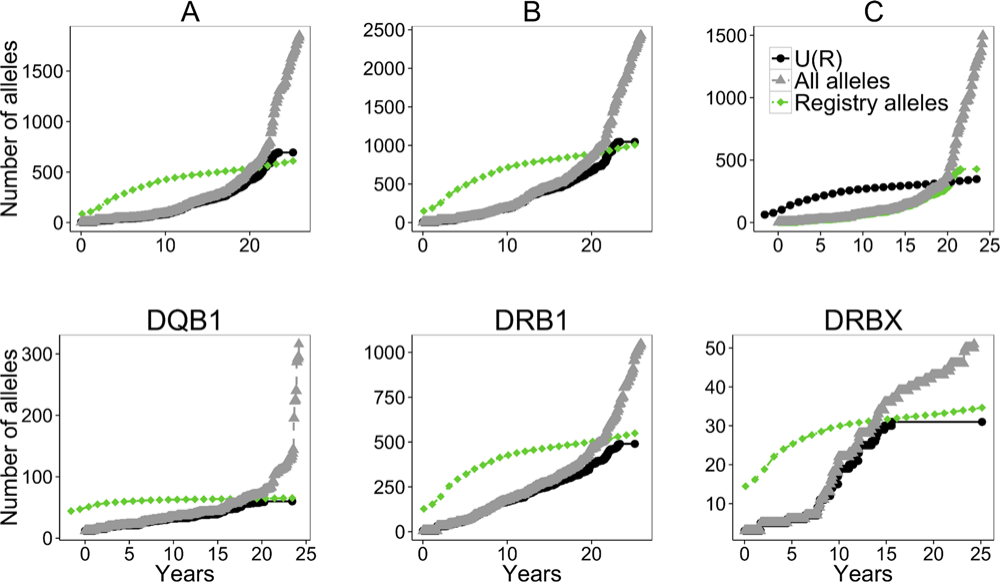
Comparison between expected and observed number of alleles for each locus. The black circles represent the numbers computed from the *U*(*R*) based estimate. The green diamonds represent the alleles in the registry, the gray triangles represent all alleles in the IMGT. The analysis is on all samples combined. Each subplot represents a different locus.

To better understand this contradiction, we computed allele sample sizes during the years 1987–2011 and calculated the expected number of alleles based on these sample sizes, using the α, *X*_*min*_ and *X*_*max*_ values obtained for the largest sample. We plotted the expected number of alleles as a function of time and compared it to the number of alleles which were identified by that date in the IMGT (Fig 7). In analyzing the entire US population as a whole, a comparison between the expected number of alleles *U*(*R*) and those actually observed presents a contradiction: Initially, the number of observed alleles was significantly less than expected based upon sample size; presently, it is much larger than expected.

The explanation for this contradiction appears to be two-fold: First, false allele discovery represents a known source of error common with PCR when crossover products occur [22] and could contribute to the observed discrepancy. Second and more importantly, the transition to sequencing-based typing (SBT) changed the fundamental nature of allele definitions and improved capacity for allele discovery. With a much larger definition of alleles and a higher discovery rate, the fundamental power law relationship would be expected to change at some point in time as the data sources move towards SBT versus older oligo or serology based methods. For example, a recent data submission from a single SBT laboratory resulted in a 30% increase in the number of observed alleles [24]. Thus, our current power-law methodology appears flawed for providing accurate estimates for the number of unique alleles and requires future modifications to accommodate the mixed data sources.

## Discussion

In this analysis, we developed a quantitative methodology for measuring haplotype and allele diversity with a particular application in mind of estimating **A** and **H** in the United States population to inform HSCT matching. Quantitative estimates of **A** and **H** are important for assessing whether typed donor cohorts have sufficient sampling depth to capture the clinically relevant HLA variation among individuals. Estimates of **A** and **H** for the Be The Match Registry suggest that current registry sampling depth is sufficient to represent only 52–100% of **A** and 21–30% of **H** across the 5 main racial populations assessed. The number of “rare” alleles and haplotypes, however, is small compared to the number of “common” ones suggesting that the current diversity of our registry is sufficient to describe 99.94–100% and 93.4–99.4% of the overall United States population with respect to allele and haplotype coverage. Further, the estimation methods developed in this article quickly converged—with limited sampling depth—to accurate estimates of allele and haplotype diversity in the presence of heavy-tailed distributions. Popular methods intended for measuring diversity with bell shaped distributions were shown to require deep samples before achieving meaningful convergence, as was the case for capture-recapture models, or failed outright to converge toward accurate estimates. A detailed comparison of our methodology with state of the art species richness estimates, as performed by the CatchAll software, show a much faster and more precise convergence to the real haplotype number using our methodology.

The methodology we developed is closely aligned with the broader fields of ecology, computation, and statistics as they relate to describing species richness, language diversity, data base storage, and occupancy. The common thread among these problems is the need to understand the behavior of “rare” events in a population with a heavy tail distribution: this can be problematic because “rare” events represent critical patterns that have minimal, if any, representation within the sample. Appropriate statistical methodologies for “rare” events recognize that light tailed distributions (often having the characteristic bell shape) are inappropriate for adequately describing population diversity. The assumption of a light tailed distribution implies that a relatively small sample is enough to detect and fully describe the population (i.e. captures all species or haplotype diversity). This intuition stems from the absence of extremely rare events, which is an untrue assumption for a heavy tail distribution. For example, in most realistic species distributions, the majority of species are very rare, and thus a very deep sample is required to detect most species. Moreover, if the tail is flat enough, another element should be incorporated, which is the extreme contribution of the most frequent species to the distribution. In such cases, most of the population will belong to a very small number of species.

Therefore, we built our model around a truncated power-law for estimating the properties of infinite discrete distributions with regularly varying heavy tails [7,8,14]. The properties of our data fit these assumptions, which require an index of variation *α* ∈ (1,2) for the PDF of frequencies: our index of variation ranged from 1.45 to 1.85 across the 21 populations. We note that to support the interpretability of our results, we modeled the PDF of frequencies whereas others have modeled the CDF of frequencies where values of *α* will be *α* - 1 units smaller. It is important to note that the convergence to the proper values of *α* occurred for a limited sample size. Thus, our method may be applicable for other surveys where the sample size is much smaller. Moreover, beyond the need to estimate the total number of different haplotype, the proposed method can be used to properly estimate *α* for small population sizes.

In the infinite case, one ignores the right hand side behavior because the “common” events offer a negligible contribution to overall diversity. Eventually, one might consider a more complex model where we define $v\left(x\right)\;=\;Cx^{-\alpha }L(x)$, with *L* (*X*) being a general function with the property that v(x) = *Cx* ^-*α*^ *L*(*x*). Such a function would account more smoothly for the different slopes in the left hand part and the right hand part of the distribution. Note however, that in realistic situations, the most frequent haplotypes (or species) are well known, and their frequency is well estimated. Thus, a simpler approach could be to split the population into frequency known haplotypes and rare haplotypes, and focus on the distribution of the rare haplotypes, taking into account that their contribution to the population coverage may be very limited, even if they constitute the vast majority of the diversity.

While we have here focused on the HLA haplotype distribution, the same formalism can be applied to other domains of ecology and population genetics, where the species richness distributions have heavy tails [25,26,27,28], see [29] for a review. In ecological scenarios, since most species are very rare, the current shrinking of the wildlife population is equivalent to a dramatic reduction in the total diversity. While in normal distributions we expect species to disappear and diversity to be significantly reduced only when the total population is close to extinction, the heavy tailed distribution of species frequencies leads to opposite conclusions. For example, assuming parameters similar to the ones obtained here, a 10 fold reduction in the total population would be equivalent to a 17–18% reduction in the number of species.

Note that in ecological scenarios, it is not appropriate to assume that all species are equally detectable at the level of the individual. Certain species are less detectable based on experimental or behavioral elements (e.g. speed of movement). A future development of such methods should be the introduction of a frequency bias in the detection probability.

Regarding validation, we compared our estimates of **A** and **H—**based upon subsamples of the data—to counts obtained by enumerating the complete data set. Although our predictions aligned reasonably with the absolute frequencies, we are concerned with the fact that the underlying data may be biased towards over-representing common alleles and haplotypes; thus, our estimates of **A** and **H** may under-represent the true population diversity. Our concern is based upon the mechanics of the EM algorithm, which trim the tail of the haplotype frequency distribution numerous times during the estimation process to generate a parsimonious and tractable candidate set; without trimming the number of potential haplotypes that greatly exceeds the number of donors. Further, expected changes in typing technology (that are already underway) from SBT to next generation sequencing (NGS) may also re-define allele definitions and increase the capacity for allele discovery. For example, NGS has the ability to detect allelic variants previously “hidden” behind genotypes that only differ by recombination [30]. In short, EM and typing technology factors could increase estimates for **A** and **H** by changing the fundamental nature of the power law relationship.

## Methods

### Haplotype and Allele Frequency Data

Six-locus high resolution HLA A~C~B~DRBX~DRB1~DQB1 (where DRBX = DRB3/4/5) haplotype frequencies were estimated across 21 race groups using an EM algorithm and 6.59 million donor HLA typings from the Be The Match Registry: complete details regarding the data and estimation are provided by Gragert [11]. In brief, an EM algorithm was utilized to resolve uncertainties in allele and chromosomal phase for a mixed resolution set of donor HLA typings (serology, sequence-specific oligonucleotide/primer, and sequence based typing). The only notable change from the Gragert methodology was that in the last iteration of the EM algorithm, a winner-take-all approach was applied where each donor contributed 1 unit of probability mass to their most likely pair of haplotypes; this is opposed to each donor assigning 1 unit of probability mass across a range of haplotype pairs—consistent with their HLA typing—in proportion to their conditional likelihood. Allele frequencies were derived as marginal sums of the haplotype frequencies.

### Methodology Validation

We have performed simulations with chosen *α* and *N*_*total*_. For each set of *α* and *N*_*total*_ values, we set *X*_min_ to be 1Ntotal, and calculated the maximum value possible for *X*_max_ using numerical solution of *x* ^*2*^(1-*x* ^α-2^)-*C* = 0 where *C* = (2-*α*)*N*_*total*_ (see Eq B6 in S2 Text). We then calculated the number of unique haplotypes expected in the total population (**H**). Next, we generated **H** *p*_*j*_ *’s* for the distribution (see S1 Text) by inverting the CDF, obtaining: pj=(Xmax1−α−Xmin1−α)rand(0,1)+[Xmin1−α]11−α where *rand*(0,1) is a uniform random number between 0 and 1. We checked that the sum of all *p*_*j*_ *’s* indeed equals 1, and very limited deviations were observed. We normalized the results to overcome the imprecise sum. We then produced a random population with these a priori probabilities, and compared the expected frequency of a haplotype unobserved in a sample (*Z*(*R*)), the expected number of unique haplotypes in the population (*U*(*R*)) and the fraction of the population covered by these haplotypes to the simulations results. Code for the estimation of *U*(*R*) and a test set are available in the Supp. Mat.

### Numerical Optimization

Given an observed population's haplotype frequency distribution, we assume that the frequency distribution is a scale free distribution with upper and lower cutoff values: *X*_min_≤*x*≤*X*_max_. We assume a zero probability of observing haplotypes with frequencies beyond these values. In order to estimate the properties of the distribution, the values of *α*, *X*_min_ and *X*_max_ should be estimated. We provide boundaries for *X*_*min*_ and *X*_*max*_ using Eq B1 and B6 in S2 Text. We then perform a numerical fit of the observed and expected (Eq 8) unique haplotypes, with a cost function of $\sum_{R'}{\left(\log(\text{U(R')-log(observed(R')}\right)}^{2}*\log(\text{observed(R'))}$ for different values of sample sizes *R* ^’^. We use an initial guess of the parameter above using Eq B1 for *X*_*min*_, the highest observed frequency *X*0_max_, and the estimate of *α*, based on Eq 9. We then extract the optimal parameters (*α*, *X*_max,_ *X*_max_), calculate the expected total number of unique haplotypes **H** and produce an estimate of the number of unique haplotypes for any population size U(R).

## Supporting Information

S1 Text Normalization for haplotype frequency density function. (DOCX)

S2 Text Range for haplotype frequency density function. (DOCX)

S3 Text Expected number of haplotypes. (DOCX)

S4 Text Calculation of probability that a haplotype is not observed and the fraction of the population covered with current observations. (DOCX)

S1 Fig Ratio of analytical estimated *U*(*R*) values divided by simulation results for different values of sample size and *α* (Inset: Analytical estimation (gray solid line) and simulation values (black circles) of U(R) (black circles) for *α* = 1.5). (TIFF)

S2 Fig Comparison of computed and observed expected number of haplotypes in a sample of size R : *U*(*R*)for four different American populations for different sample sizes R. The gray squares are observations. All estimates were performed using 10% of the sample. The lines are an estimation of *U*(*R*)with all free parameters (full purple), maximal *Xmax* and free *α* (dashed green line), free *Xmax* and free *α* but fixed *Xmin* (dashed black line) and free boundaries but *α* based on the Clauset estimator (double dashed red line).
(TIFF)

S3 Fig Haplotype relative frequency distribution for the African-American, Japanese, Black Caribbean and South/Central American Hispanic populations. Observations are in gray dashed lines with triangles, and black solid lines with circles are the linear regressions.
(TIFF)

S4 Fig Haplotype relative frequency distribution for the African, Korean, Caribbean Hispanic and Black South or Central America populations. Observations are in gray dashed lines with triangles, and black solid lines with circles are the linear regressions.
(TIFF)

S5 Fig Haplotype relative frequency distribution for the Other Southeast Asian, Caribbean Indian, South Asian and MidEast/North Coast of Africa populations. Observations are in gray dashed lines with triangles, and black solid lines with circles are the linear regressions.
(TIFF)

S6 Fig Haplotype relative frequency distribution for the American Indian South or Central America, Mexican or Chicano, European American and Vietnamese populations. Observations are in gray dashed lines with triangles, and black solid lines with circles are the linear regressions.
(TIFF)

S7 Fig Haplotype relative frequency distribution for Alaska Native or Aleut, North American Indian, Filipino and Hawaiian or other Pacific Islander populations. Observations are in gray dashed lines with triangles, and black solid lines with circles are the linear regressions.
(TIFF)

S8 Fig Haplotype relative frequency distribution for the Chinese population. Observations are in gray dashed lines with triangles, and black solid lines with circles are the linear regressions.
(TIFF)

S9 Fig Ratio of analytical estimated fraction of covered population values divided by simulation results for different values of sample size and *α* (Inset: Analytical estimation (gray solid line) and simulation values (black circles) of fraction of covered population for *α* = 1.5.). (TIFF)

S10 Fig Ratio of analytical estimated values of the probability that there exists at least one unobserved haplotype divided by simulations results for different values of sample size and *α* (Inset: Analytical estimation (gray solid line) and simulation values (black circles) of the probability that there exists at least one unobserved haplotype for *α* = 1.5.). (TIFF)

S1 Code Matlab code for estimation of U(R). (ZIP)

## References

[1] Dunn GP, Old LJ, Schreiber RD (2004) The immunobiology of cancer immunosurveillance and immunoediting. Immunity 21: 137–148. 15308095 10.1016/j.immuni.2004.07.017

[2] Hansen J, Yamamoto K, Petersdorf E, Sasazuki T (1998) The role of HLA matching in hematopoietic cell transplantation. Reviews in immunogenetics 1: 359–373. 11256427

[3] Petersdorf EW, Anasetti C, Martin PJ, Gooley T, Radich J, et al (2004) Limits of HLA mismatching in unrelated hematopoietic cell transplantation. Blood 104: 2976–2980. 15251989 10.1182/blood-2004-04-1674

[4] Kollman C, Maiers M, Gragert L, Müller C, Setterholm M, et al (2007) Estimation of HLA-A,-B,-DRB1 haplotype frequencies using mixed resolution data from a national registry with selective retyping of volunteers. Human immunology 68: 950–958. 10.1016/j.humimm.2007.10.009 18191722

[5] Friedman JH (1997) On bias, variance, 0/1—loss, and the curse-of-dimensionality. Data mining and knowledge discovery 1: 55–77.

[6] Glanville J, Zhai W, Berka J, Telman D, Huerta G, et al (2009) Precise determination of the diversity of a combinatorial antibody library gives insight into the human immunoglobulin repertoire. Proceedings of the National Academy of Sciences 106: 20216–20221. 10.1073/pnas.0909775106 19875695 PMC2787155

[7] Ohannessian MI, Dahleh MA (2012) Rare Probability Estimation under Regularly Varying Heavy Tails. Journal of Machine Learning Research-Proceedings Track 23: 21.21–21.24.

[8] Gnedin A, Hansen B, Pitman J (2007) Notes on the occupancy problem with infinitely many boxes: general asymptotics and power laws. Probab Surv 4: 146–171.

[9] Robinson J, Waller MJ, Parham P, de Groot N, Bontrop R, et al (2003) IMGT/HLA and IMGT/MHC: sequence databases for the study of the major histocompatibility complex. Nucleic acids research 31: 311–314. 12520010 10.1093/nar/gkg070 PMC165517

[10] Mack S, Cano P, Hollenbach J, He J, Hurley C, et al (2013) Common and well‐documented HLA alleles: 2012 update to the CWD catalogue. Tissue antigens 81: 194–203. 10.1111/tan.12093 23510415 PMC3634360

[11] Gragert L, Madbouly A, Freeman J, Maiers M (2013) Six-locus high resolution HLA haplotype frequencies derived from mixed-resolution DNA typing for the entire US donor registry. Human immunology 74: 1313–1320. 10.1016/j.humimm.2013.06.025 23806270

[12] Klitz W, Hedrick P, Louis EJ (2012) New reservoirs of HLA alleles: pools of rare variants enhance immune defense. Trends in Genetics 28: 480–486. 10.1016/j.tig.2012.06.007 22867968

[13] Cooke RM, Nieboer D (2011) Heavy-Tailed Distributions: Data, Diagnostics, and New Developments. Resources for the Future Discussion Paper.

[14] Gnedin A, Iksanov A, Marynych A (2010) The Bernoulli sieve: an overview. arXiv preprint arXiv:10055705.

[15] Adamic LA (2000) Zipf, power-laws, and pareto-a ranking tutorial. Xerox Palo Alto Research Center, Palo Alto, CA, http://ginger hpl hp com/shl/papers/ranking/ranking html.

[16] Clauset A, Shalizi CR, Newman ME (2009) Power-law distributions in empirical data. SIAM review 51: 661–703.

[17] Muniruzzaman A (1957) On measures of location and dispersion and tests of hypotheses on a Pareto population. Bulletin of the Calcuta Statistical Association 7: 115–123.

[18] Karlin S (1967) Central limit theorems for certain infinite urn schemes. J Math Mech 17: 373–401.

[19] Gotelli NJ, Colwell RK (2011) Estimating species richness Biological diversity: frontiers in measurement and assessment: 39–54.

[20] ter Steege H, Pitman NC, Sabatier D, Baraloto C, Salomão RP, et al (2013) Hyperdominance in the Amazonian tree flora. Science 342: 1243092 10.1126/science.1243092 24136971

[21] Bunge J, Woodard L, Böhning D, Foster JA, Connolly S, et al (2012) Estimating population diversity with CatchAll. Bioinformatics 28: 1045–1047. 10.1093/bioinformatics/bts075 22333246 PMC3315724

[22] Holcomb C, Rastrou M, Williams T, Goodridge D, Lazaro A, et al (2014) Next-generation sequencing can reveal in vitro-generated PCR crossover products: some artifactual sequences correspond to HLA alleles in the IMGT/HLA database. Tissue antigens 83: 32–40. 10.1111/tan.12269 24355006

[23] Ewens WJ (1972) The sampling theory of selectively neutral alleles. Theoretical population biology 3: 87–112. 4667078 10.1016/0040-5809(72)90035-4

[24] Hernández-Frederick C, Giani A, Cereb N, Sauter J, Silva-González R, et al (2014) Identification of 2127 new HLA class I alleles in potential stem cell donors from Germany, the United States and Poland. Tissue antigens 83: 184–189. 10.1111/tan.12304 24571476 PMC4199310

[25] Magurran AE (1988) Ecological diversity and its measurement: Springer.

[26] Barabasi A-L, Oltvai ZN (2004) Network biology: understanding the cell's functional organization. Nature Reviews Genetics 5: 101–113. 14735121 10.1038/nrg1272

[27] Koonin EV, Wolf YI, Karev GP (2002) The structure of the protein universe and genome evolution. Nature 420: 218–223. 12432406 10.1038/nature01256

[28] Huynen MA, Van Nimwegen E (1998) The frequency distribution of gene family sizes in complete genomes. Molecular biology and evolution 15: 583–589. 9580988 10.1093/oxfordjournals.molbev.a025959

[29] Koonin EV (2011) Are there laws of genome evolution? PLoS computational biology 7: e1002173 10.1371/journal.pcbi.1002173 21901087 PMC3161903

[30] Lind C, Ferriola D, Mackiewicz K, Papazoglou A, Sasson A, et al (2013) Filling the gaps–The generation of full genomic sequences for 15 common and well-documented HLA class I alleles using next-generation sequencing technology. Human immunology 74: 325–329. 10.1016/j.humimm.2012.12.007 23246585

