# Supplementary material for: Power Laws for Heavy-Tailed Distributions: Modeling Allele and Haplotype Diversity for the National Marrow Donor Program
Source: PLoS Comput Biol. 2015 Apr 22;11(4):e1004204. doi: 10.1371/journal.pcbi.1004204 (PMC4406525; doi:10.1371/journal.pcbi.1004204)
Supplement: S1 Text — (DOCX) [file pcbi.1004204.s001.docx]

## Text S1. Normalization for haplotype frequency density function

We assume a population of size $N_{total}$ with $H$haplotypes in the population, and an *a priori* probability of $p_{j}$ for haplotype j $(1\leq j\leq H,0\leq p_{j}\leq1)$. The sum of these probabilities $(\sum_{i=1}^{H} p_{j})$is 1, where $H$ is the total number of haplotypes.

We then use a continuous approximation, and define the total number of $p_{j}$ values in the interval $[x,x+dx]$to be$\mu_{H}(x)dx$. We also define a probability density function $p_{H}(x)=\frac{\mu_{H}(x)}{H}$ and assume it fits a truncated power law function: $p_{H}(x)=Cx^{-\alpha}$.This distribution cannot be in the entire range [0,1], since it can diverge for low enough values of $x$. We thus assume a limited range of allowed$p_{j}$. This range differs for different values of $\alpha$ and $N_{total}$. Formally, $p_{H}(x)\neq0,X_{\min}\leq x\leq X_{\max}$.$X_{\min}$ is the smallest possible probability for a haplotype and $X_{\max}$ is the largest possible probability for a haplotype.

In this notation, $(\sum_{i=1}^{H} p_{j})=1$ is approximated by the integral:

(A1) $\int_{X_{\min}}^{X_{\max}} x\mu_{H}(x)dx=1.$

This translates to:

(A2) $\int_{X_{\min}}^{X_{\max}} xp_{H}(x)dx=\frac{1}{H}$,

with the standard normalization of:

(A3) $\int_{X_{\min}}^{X_{\max}} p_{H}(x)dx=1.$

(A4) $\int_{X_{\min}}^{X_{\max}} Cx^{1-\alpha}dx=\left.\frac{Cx^{2-\alpha}}{2-\alpha}\right|_{X_{\min}}^{X_{\max}}=\frac{C(X_{\max}^{2-\alpha}-X_{\min}^{2-\alpha})}{2-\alpha}=\frac{1}{H}$,

and

(A5) $\int_{X_{\min}}^{X_{\max}} Cx^{-\alpha}dx=\left.\frac{Cx^{1-\alpha}}{1-\alpha}\right|_{X_{\min}}^{X_{\max}}=\frac{C(X_{\max}^{1-\alpha}-X_{\min}^{1-\alpha})}{1-\alpha}=1.$

Given $X_{\max}$and $X_{\min}$, the normalization factor is:

(A6) $C=\frac{1}{H}\frac{2-\alpha}{X_{\max}^{2-\alpha}-X_{\min}^{2-\alpha}}$

leading to:

(A7) $H=\frac{2-\alpha}{1-\alpha}\frac{X_{\max}^{1-\alpha}-X_{\min}^{1-\alpha}}{X_{\max}^{2-\alpha}-X_{\min}^{2-\alpha}}$.
