# Supplementary material for: Power Laws for Heavy-Tailed Distributions: Modeling Allele and Haplotype Diversity for the National Marrow Donor Program
Source: PLoS Comput Biol. 2015 Apr 22;11(4):e1004204. doi: 10.1371/journal.pcbi.1004204 (PMC4406525; doi:10.1371/journal.pcbi.1004204)
Supplement: S2 Text — (DOCX) [file pcbi.1004204.s002.docx]

## Text S2. Range for haplotype frequency density function

We can either assume that the power law distribution is on the whole range possible [$X_{\min}$, $X_{\max}$] and that the upper cut $X_{\max}$will be determined by the most frequent haplotype, or we can assume that the power law distribution is on a sub range of [$X_{\min}$, $X_{\max}$] , where $X_{\min}$ and $X_{\max}$are free parameter bound by the maximal possible range defined below. Assuming the first case, we can estimate $X_{\min}$and $X_{\max}$ the following way:

First we note that the sampled haplotype cannot have an absolute frequency which is smaller than 1. We thus first approximate that the minimal relative frequency is:

(B1) $X_{\min}\geq\frac{1}{N_{total}}$ .

The minimal difference between the relative frequencies of existing haplotypes is $\frac{1}{N_{total}}$, and the most frequent haplotype is typically unique in its bin, leading to an upper estimate of $X_{\max}$as:

(B2) $v_{H}(X_{\max})=1$

or:

(B3) $p_{H}(X_{\max})=\frac{N_{total}}{H}.$

Introducing $p_{H}(x)=Cx^{-\alpha}$leads to:

(B4) $CX_{\max}^{-\alpha}=\frac{N_{total}}{H}.$

Using the assumption that $X_{\max}$is at is upper bound as determined by $N_{total}$, leads to:

(B5) $\begin{matrix} \frac{C(X_{\max}^{2-\alpha}-X_{\min}^{2-\alpha})}{(2-\alpha)CX_{\max}^{-\alpha}}=\frac{1}{N_{total}}\Rightarrow\frac{(X_{\max}^{2-\alpha}-X_{\min}^{2-\alpha})}{X_{\max}^{-\alpha}}=\frac{(2-\alpha)}{N_{total}} \\ \Rightarrow\left(\frac{X_{\max}}{X_{\min}}\right)^{2}-\left(\frac{X_{\max}}{X_{\min}}\right)^{\alpha}=\frac{(2-\alpha)}{X_{\min}^{2}N_{total}} \end{matrix}$,

which can be solved numerically, by using $X_{\min}=1/N_{total}$ and setting $y=N_{total}X_{\max}$ to obtain:

(B6) $y^{2}-y^{\alpha}=(2-\alpha)N_{total}$.

The total number of unique haplotypes, as shown in Eq. 8, is:

(B7) $H=\frac{2-\alpha}{1-\alpha}\frac{X_{\max}^{1-\alpha}-X_{\min}^{1-\alpha}}{X_{\max}^{2-\alpha}-X_{\min}^{2-\alpha}}$

Together with (B5) it can be computed:

(B8) $H=\frac{N_{total}}{(1-\alpha)}\left[X_{\max}-\frac{(X_{\min}^{1-\alpha})}{X_{\max}^{-\alpha}}\right]$.

In principle, in the case of$\alpha<<2,C=\frac{(2-\alpha)N_{total}}{H}>>0$, if we neglect the lower boundary, we obtain the first order approximation of:

(B9) $X_{\max}\approx\sqrt{\frac{2-\alpha}{N_{total}}}$.

Introducing this estimate into (B5) yields the second order approximation:

(B10) $X_{\max}^{2}=\frac{(2-\alpha)}{N_{total}}-\frac{(X_{\min}^{2-\alpha})}{X_{\max}^{-\alpha}}\Rightarrow X_{\max}\approx\sqrt{\frac{(2-\alpha)}{N_{total}}+\frac{(X_{\min}^{2-\alpha})}{\sqrt{\frac{2-\alpha}{N_{total}}}^{-\alpha}}}$.

This approximation can be used instead of the implicit solution above. However, we did not use this approximation in our analysis. Instead, we used an upper cutoff based on Eq. B6 and used $X_{\max}$as a free parameter.
