# Supplementary material for: Power Laws for Heavy-Tailed Distributions: Modeling Allele and Haplotype Diversity for the National Marrow Donor Program
Source: PLoS Comput Biol. 2015 Apr 22;11(4):e1004204. doi: 10.1371/journal.pcbi.1004204 (PMC4406525; doi:10.1371/journal.pcbi.1004204)
Supplement: S3 Text — (DOCX) [file pcbi.1004204.s003.docx]

## Text S3. Expected number of haplotypes

The total number of haplotypes in a sample is given by:

(C1) $H=\frac{1}{\int_{X_{\min}}^{X_{\max}} xp_{H}(x)dx}.$

We define U(R) to be the total number of different haplotypes expected to be discovered in a sample of size *R*.

The probability of not sampling haplotype *j* with probability $p_{j}$ in a sample of size *R* is $(1-p_{j})^{R}$. Therefore, the probability of observing this haplotype at least once is$1-(1-p_{j})^{R}$.We term this the “discovery” probability. Thus, using the properties of expectation the number of haplotypes discovered out of the total number of possible haplotypes **H** is:

(C2) $U(R)=\sum_{j} p(Haplotype\text{ }j\text{ }observed\text{ }at\text{ }least\text{ }once\text{)}.=\sum_{j} 1-(1-p_{j})^{R}.$

In the continuous approximation this converges to:

(C3)

$U\left(R\right)=\int_{X_{\min}}^{X_{\max}} (1-(1-x)^{R})\mu_{H}(x)dx=H-\frac{2-\alpha}{X_{\max}^{2-\alpha}-X_{\min}^{2-\alpha}}R^{\alpha-1}\left[\gamma(1-\alpha,RX_{\max})-\gamma(1-\alpha,RX_{\min})\right]$,where $\gamma$ is the incomplete gamma function.
