# Supplementary material for: Power Laws for Heavy-Tailed Distributions: Modeling Allele and Haplotype Diversity for the National Marrow Donor Program
Source: PLoS Comput Biol. 2015 Apr 22;11(4):e1004204. doi: 10.1371/journal.pcbi.1004204 (PMC4406525; doi:10.1371/journal.pcbi.1004204)
Supplement: S4 Text — (DOCX) [file pcbi.1004204.s004.docx]

## Text S4. Calculation of the probability that a haplotype is not observed and of the fraction of the population covered with current observations

We estimate the probability that a person in the total population has a haplotype that is not present in a sample of size R – $Z(R)$. This can be computed by first computing the probability that a given haplotype *j* is not observed in a population of size R, using the Poisson approximation:

(D1) $p(\text{haplotype }j\text{ is not observed in a sample of size }R)=e^{-p_{j}R}$ .

The probability that a haplotype from a random person in the population is haplotype *j* and that this haplotype is not observed in a sample of size R is: $p_{j}e^{-p_{j}R}$.

One can thus compute Z(R) to be the sum of $p_{j}e^{-p_{j}R}$over all haplotypes

(D2) $Z(R)=\sum_{j} p_{j}e^{-p_{j}R}=E(e^{-p_{j}R})$.

In order to explicitly compute$Z(R)$, assume an initial a priori probability density of$p_{H}(x)$, as defined by Eq. 5, and a total population of $N{}_{total}$haplotypes. We can then approximate Eq. D2 by:

(D3) $Z(R)=\int_{X_{\min}}^{X_{\max}} p_{H}(x)xe^{-xR}dx$.

If one assumes a power law distribution: $p_{H}(x)=Cx^{-\alpha};X_{\min}\leq x\leq X_{\max}$ , one obtains:

(D4) $Z(R)=\int_{X_{\min}}^{X_{\max}} Cx^{1-\alpha}e^{-xR}dx=\frac{2-\alpha}{X_{\max}^{2-\alpha}-X_{\min}^{2-\alpha}}R^{\alpha-2}[\gamma(2-\alpha,RX_{\max})-\gamma(2-\alpha,RX_{\min})]$ ,

where $\gamma$ is the incomplete gamma function.

The fraction of the population which is covered is $1-Z(R)$, leading to:

(D5) $\begin{matrix} FractionCovered(R)=1-\int_{X_{\min}}^{X_{\max}} p_{H}(x)xe^{-xR}dx= \\ =1-\frac{2-\alpha}{X_{\max}^{2-\alpha}-X_{\min}^{2-\alpha}}R^{\alpha-2}[\gamma(2-\alpha,RX_{\max})-\gamma(2-\alpha,RX_{\min})] \end{matrix}$ ,

where $\gamma$ is again the incomplete gamma function.
