# Supplementary figures and images for: Power Laws for Heavy-Tailed Distributions: Modeling Allele and Haplotype Diversity for the National Marrow Donor Program
Source: PLoS Comput Biol. 2015 Apr 22;11(4):e1004204. doi: 10.1371/journal.pcbi.1004204 (PMC4406525; doi:10.1371/journal.pcbi.1004204)

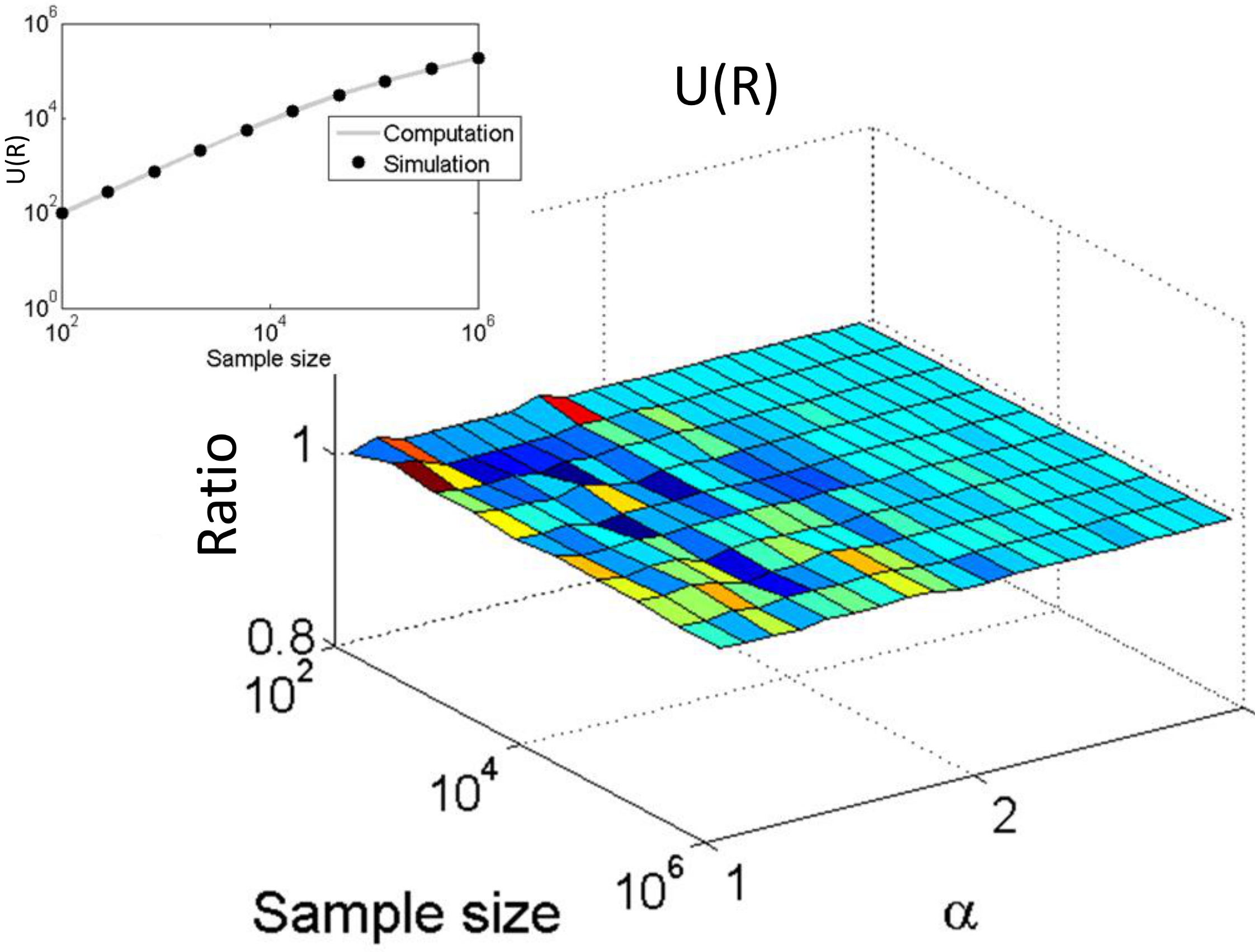

Supplement: S1 Fig — (TIFF) [file pcbi.1004204.s005.tiff]

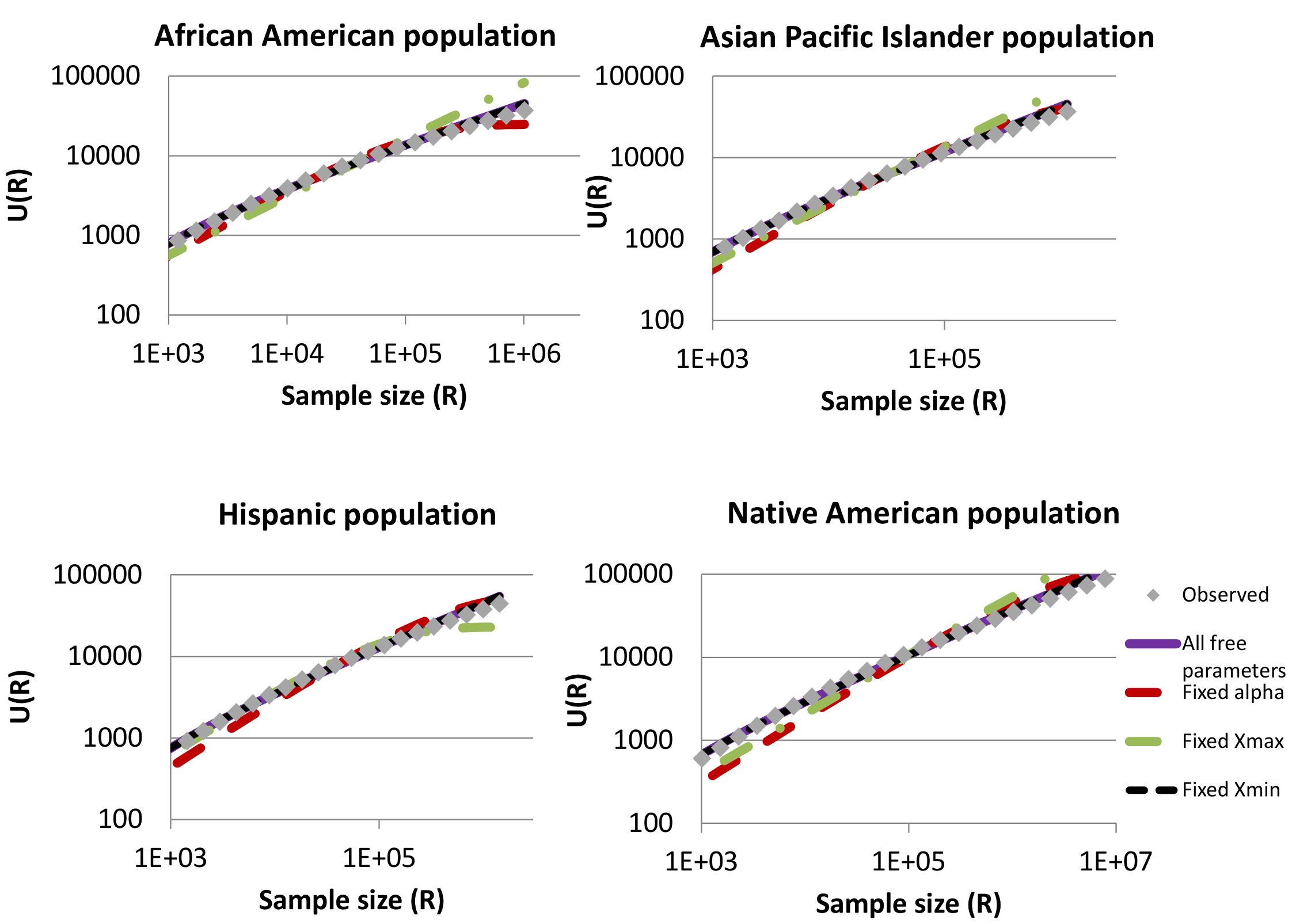

Supplement: S2 Fig — : U(R)for four different American populations for different sample sizes R. The gray squares are observations. All estimates were performed using 10% of the sample. The lines are an estimation of U(R)with all free parameters (full purple), maximal Xmax and free α (dashed green line), free Xmax and free α but fixed Xmin (dashed black line) and free boundaries but α based on the Clauset estimator (double dashed red line). (TIFF) [file pcbi.1004204.s006.tiff]

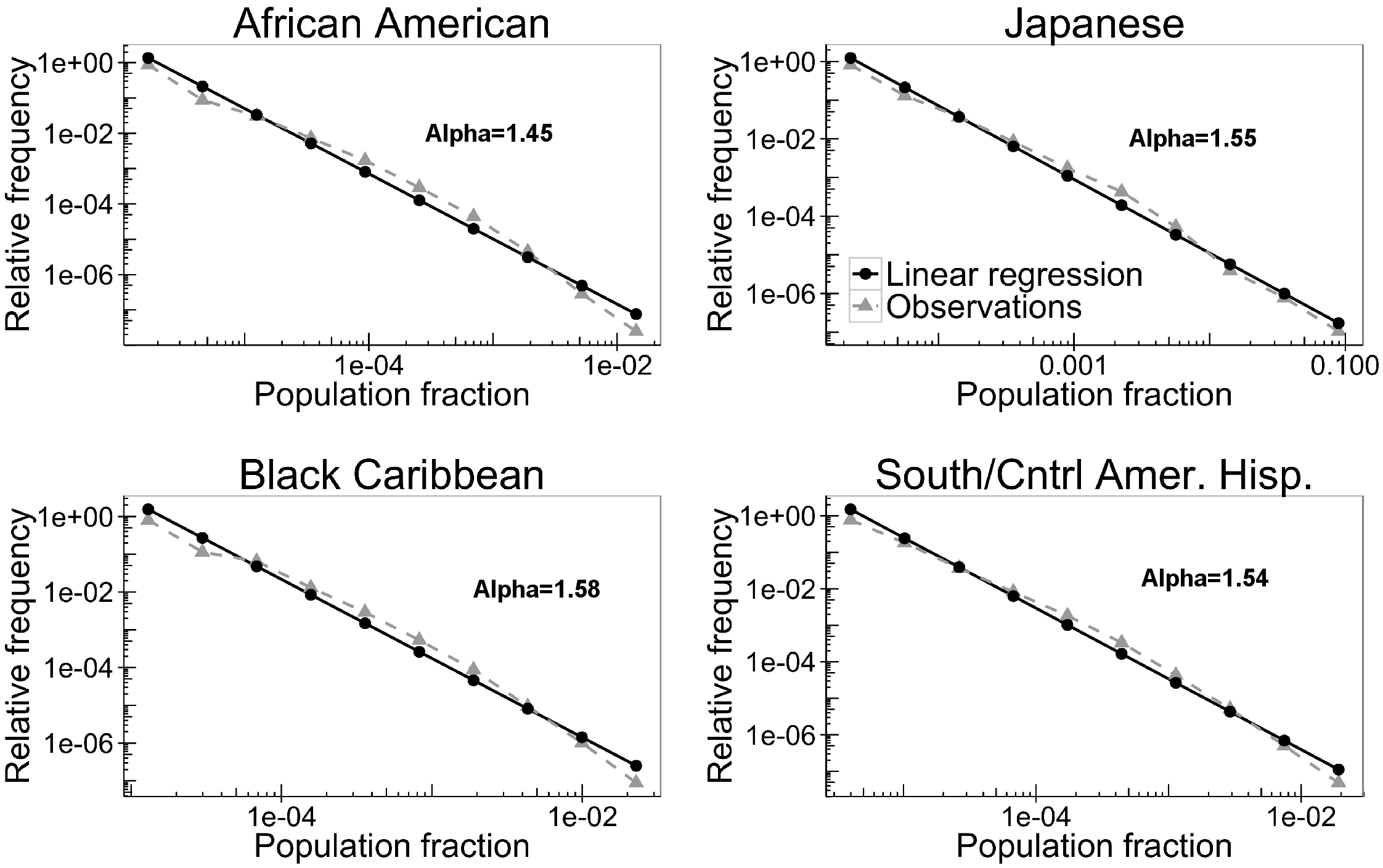

Supplement: S3 Fig — Observations are in gray dashed lines with triangles, and black solid lines with circles are the linear regressions. (TIFF) [file pcbi.1004204.s007.tiff]

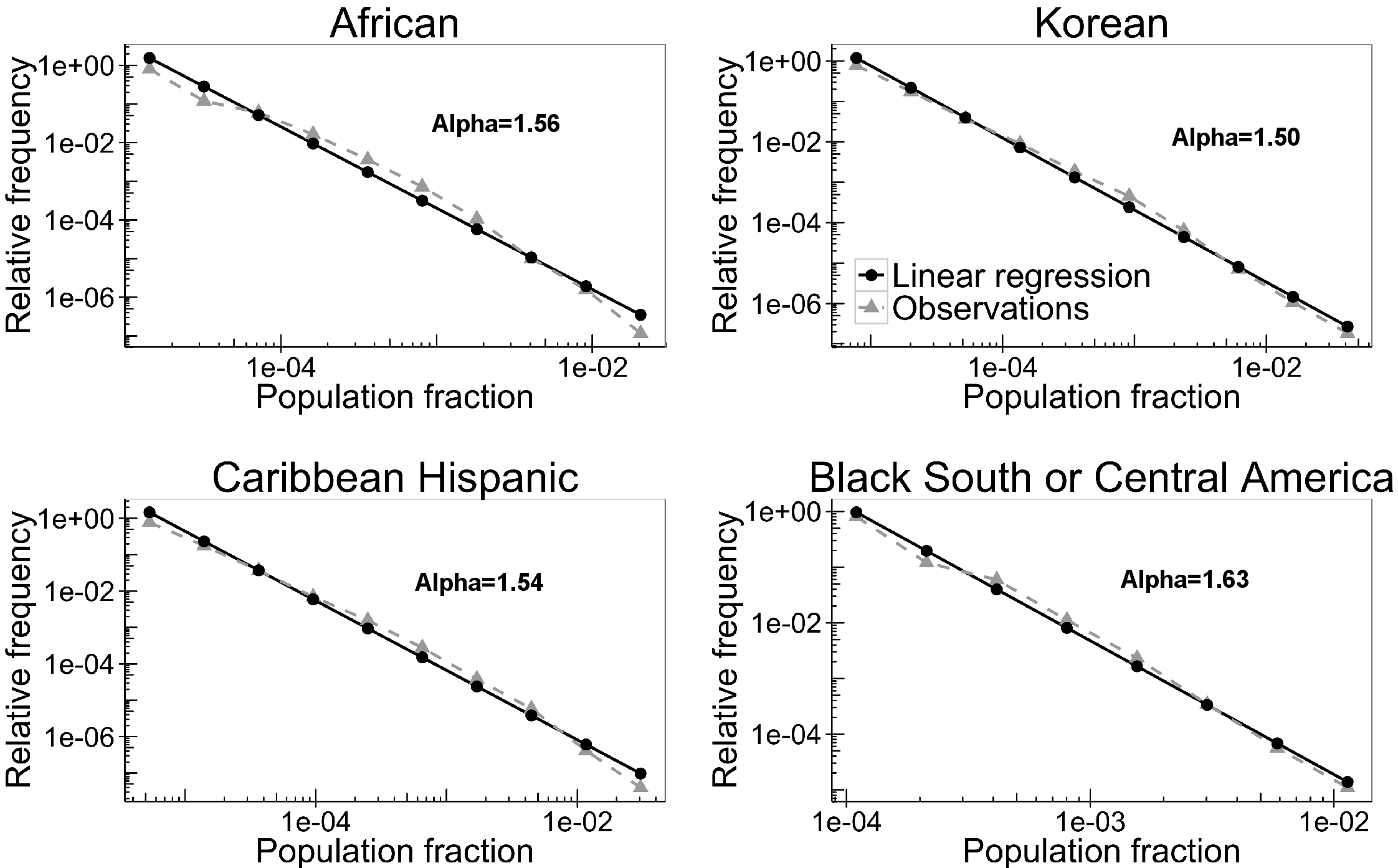

Supplement: S4 Fig — Observations are in gray dashed lines with triangles, and black solid lines with circles are the linear regressions. (TIFF) [file pcbi.1004204.s008.tiff]

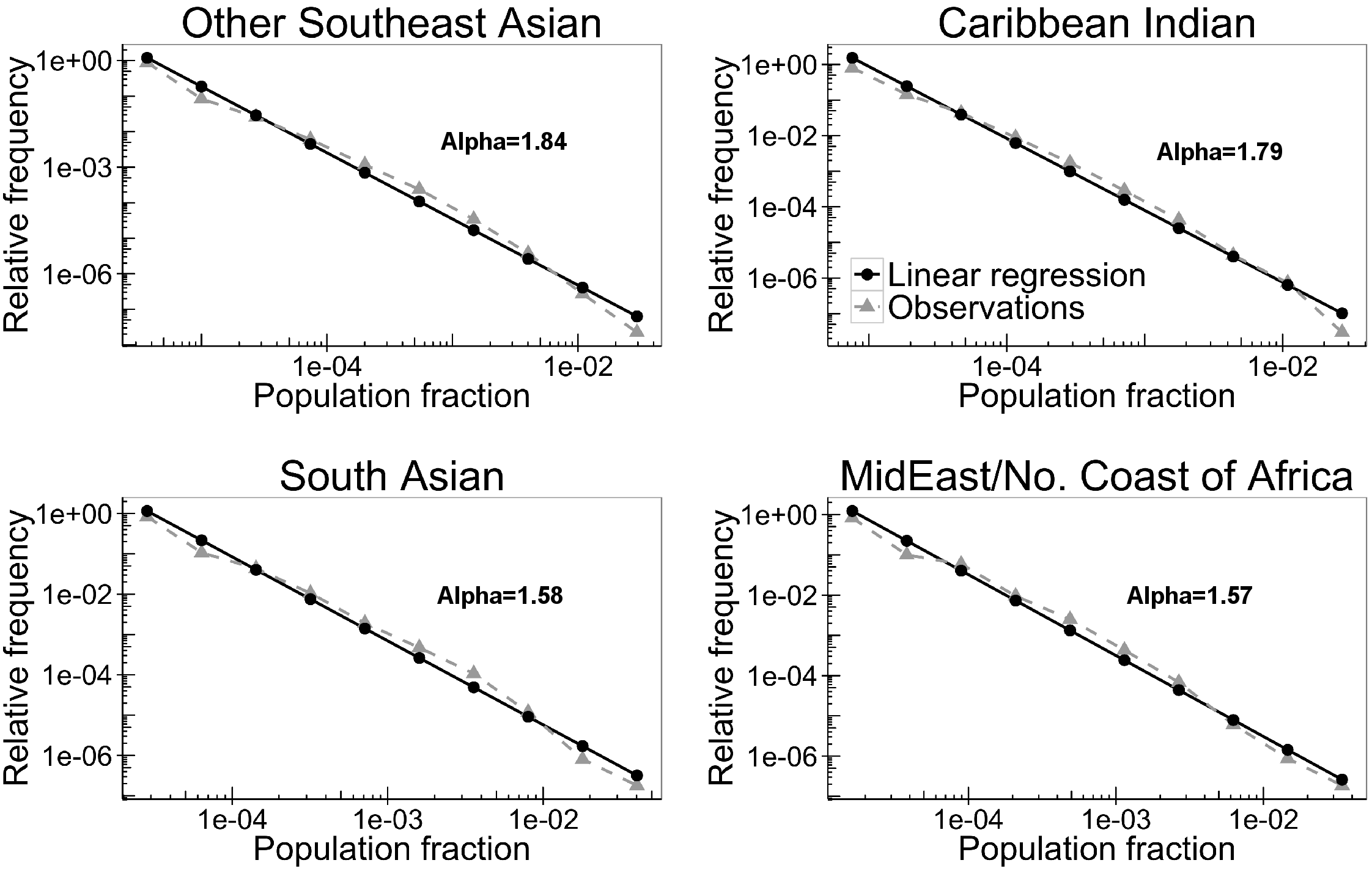

Supplement: S5 Fig — Observations are in gray dashed lines with triangles, and black solid lines with circles are the linear regressions. (TIFF) [file pcbi.1004204.s009.tiff]

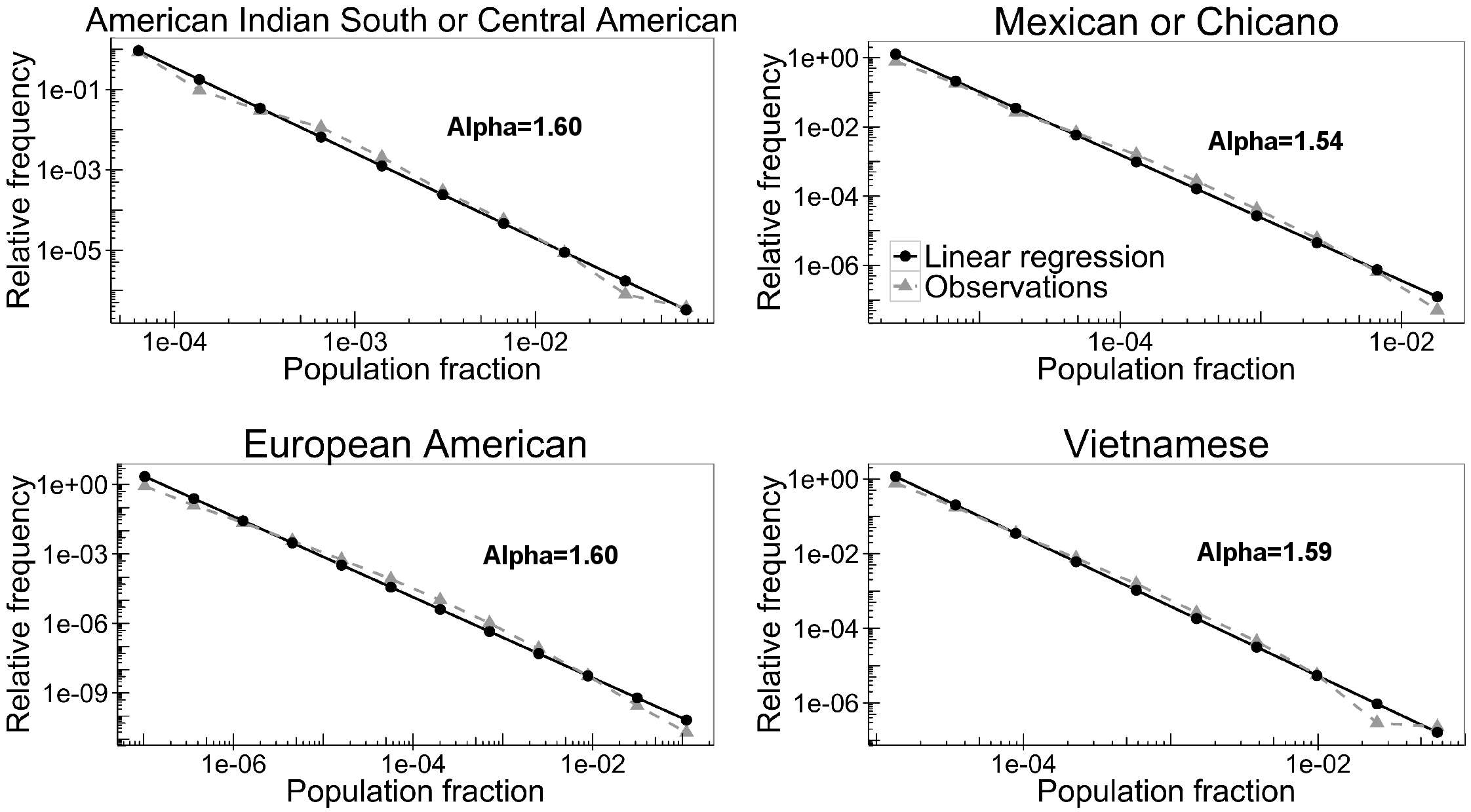

Supplement: S6 Fig — Observations are in gray dashed lines with triangles, and black solid lines with circles are the linear regressions. (TIFF) [file pcbi.1004204.s010.tiff]

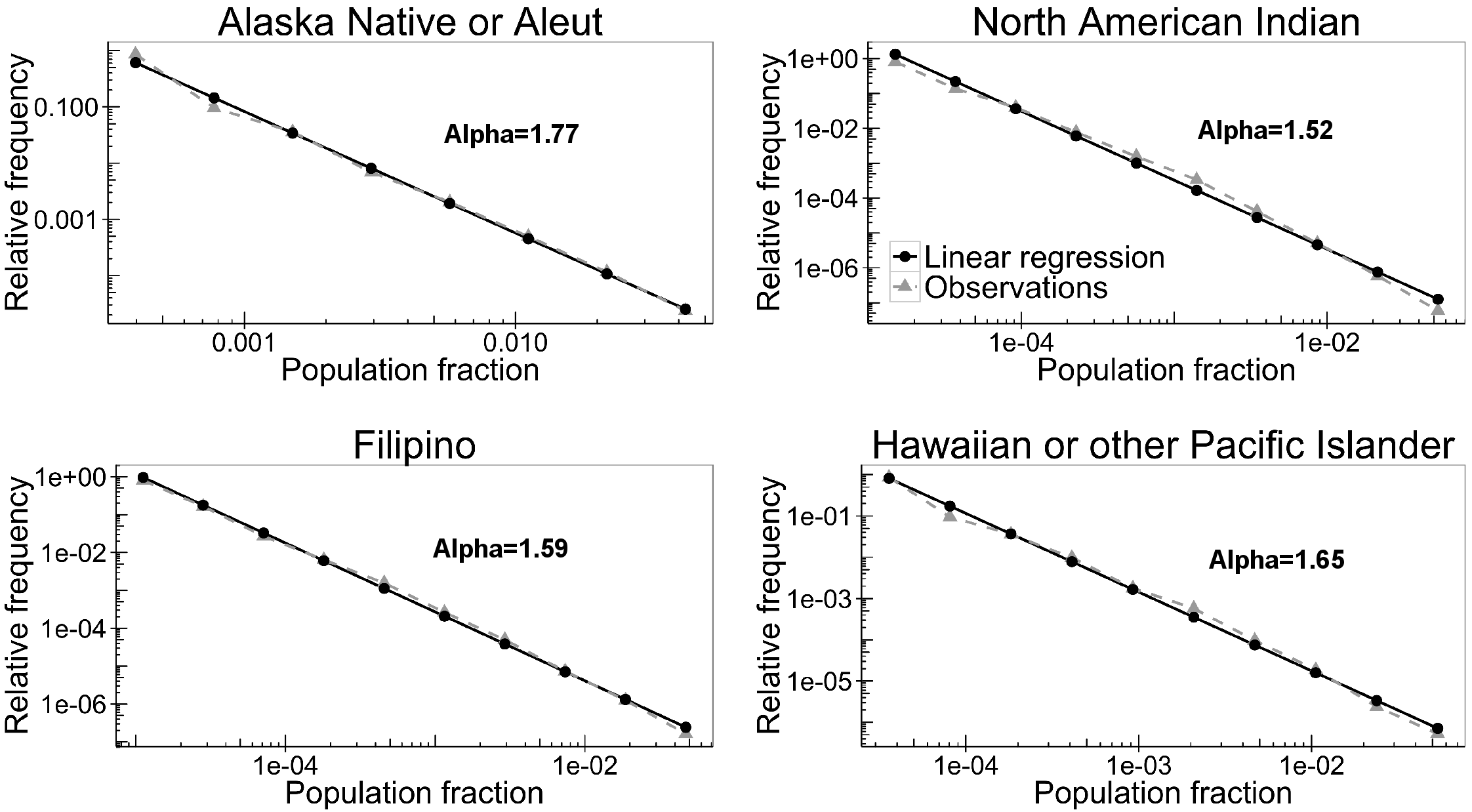

Supplement: S7 Fig — Observations are in gray dashed lines with triangles, and black solid lines with circles are the linear regressions. (TIFF) [file pcbi.1004204.s011.tiff]

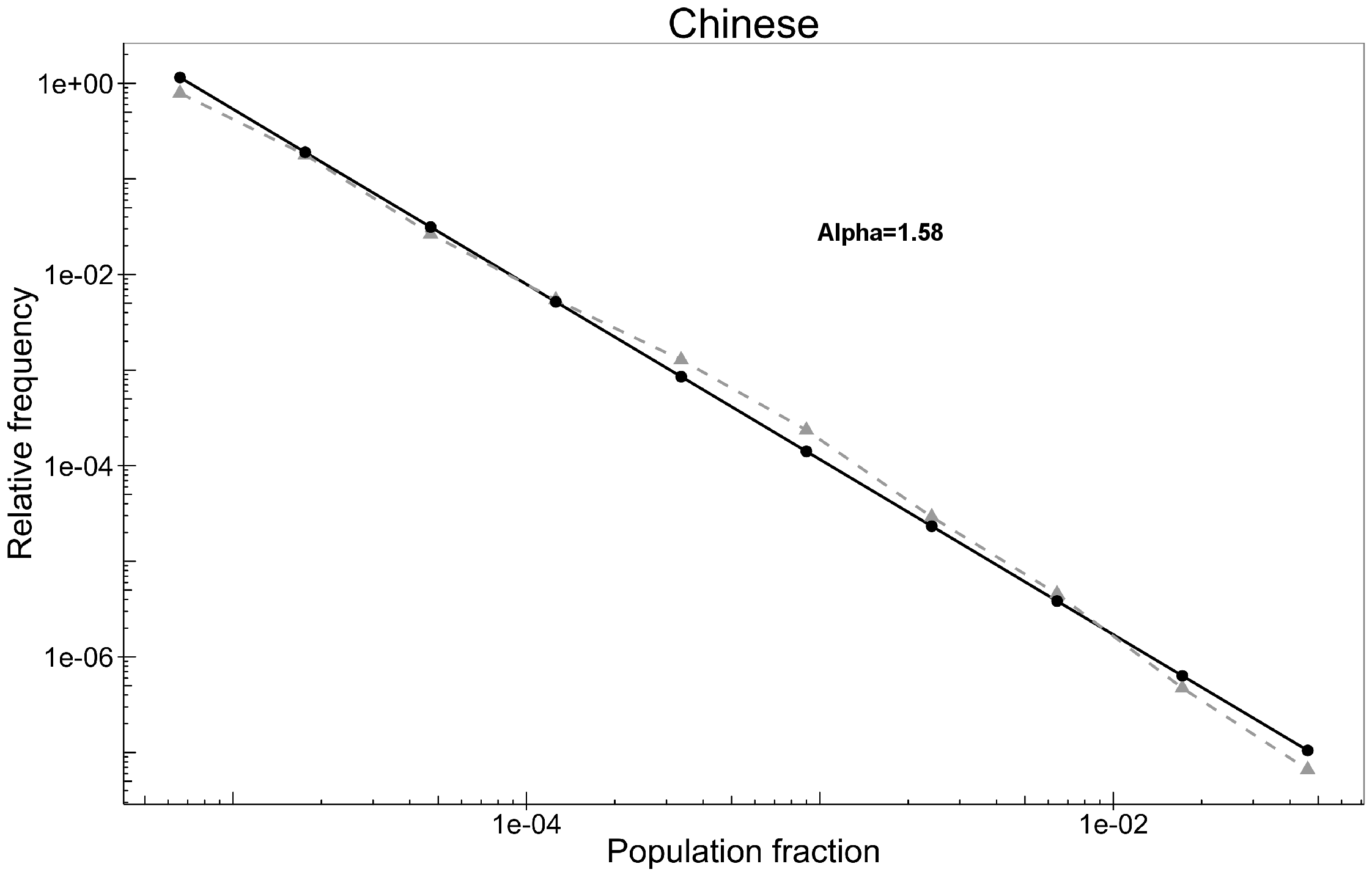

Supplement: S8 Fig — Observations are in gray dashed lines with triangles, and black solid lines with circles are the linear regressions. (TIFF) [file pcbi.1004204.s012.tiff]

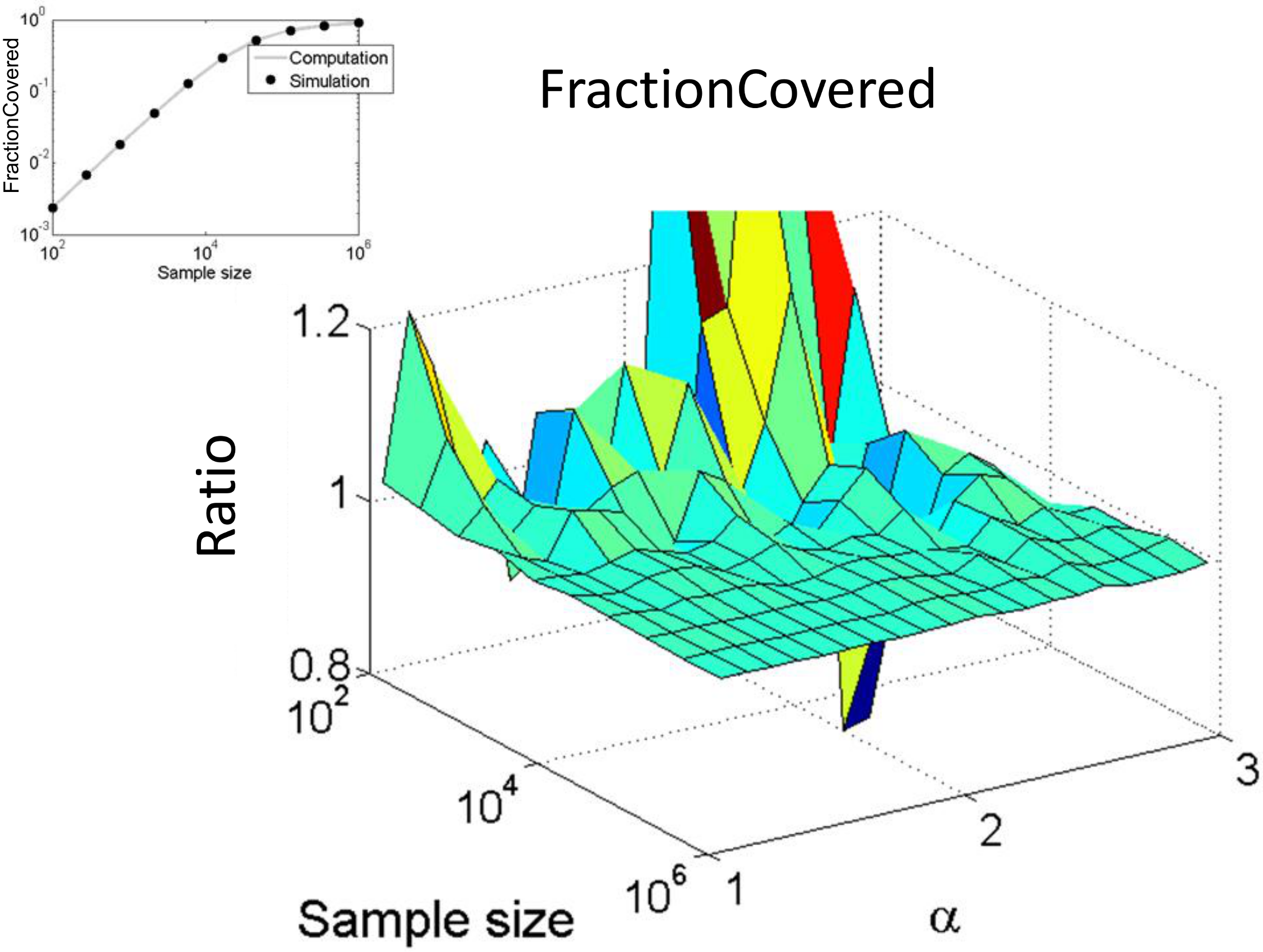

Supplement: S9 Fig — (TIFF) [file pcbi.1004204.s013.tiff]

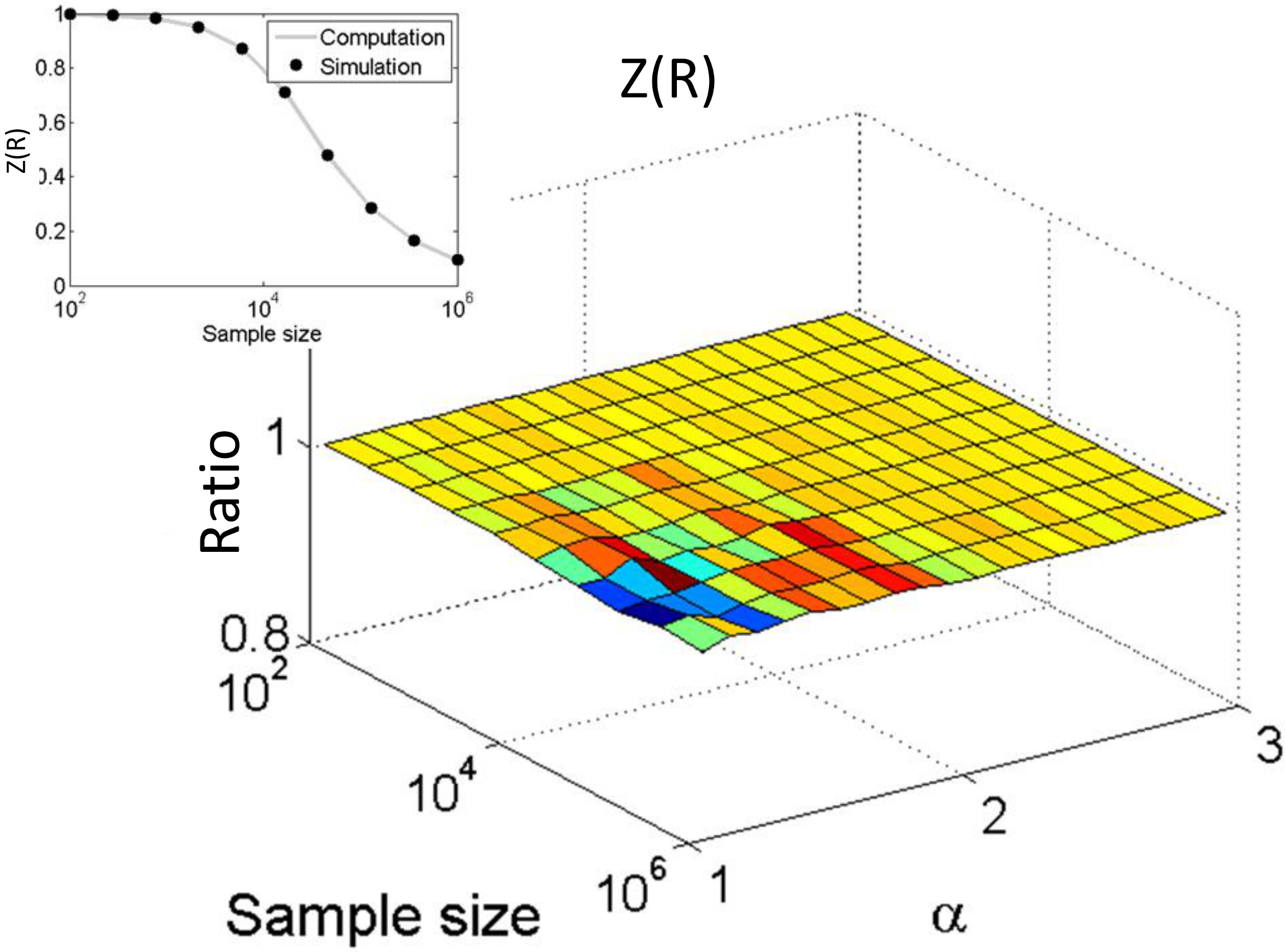

Supplement: S10 Fig — (TIFF) [file pcbi.1004204.s014.tiff]
